# Engineering Nitrogen Vacancies in Polymeric Carbon Nitride to Promote Interfacial Electron Transfer for Visible‐Light Driven Hydrogen Evolution

**DOI:** 10.1002/smll.75195

**Published:** 2026-08-21

**Authors:** Zeyi Zhang, Han Zhao, Lingshen Meng, Yu Wang, Fei Guo, Carlos A. Triana, Yonggui Zhao, Jingguo Li, Xiaoyuan Liu, Greta R. Patzke

**Affiliations:** ^1^ Department of Chemistry University of Zurich Zurich Switzerland; ^2^ Department of Chemical Engineering University College London London United Kingdom; ^3^ State Key Laboratory of Advanced Environmental Technology School of Environment University of Science and Technology of China Hefei China; ^4^ Department of Chemistry and Key Laboratory for Preparation and Application of Ordered Structural Materials of Guangdong Shantou University Shantou Guangdong China

**Keywords:** DFT calculations, electron transfer, g‐C_3_N_4_, nitrogen vacancies, photocatalytic hydrogen evolution, structure‐activity relationships

## Abstract

Polymeric carbon nitride (g‐C_3_N_4_, GCN) is a highly promising metal‐free photocatalyst for solar hydrogen production. However, its activity is still constrained by insufficient visible‐light absorption and poor interlayer charge transport. Defect engineering of GCN has been widely deployed to promote photocatalytic hydrogen evolution efficiency, but a significant knowledge gap persists in linking specific defect structures to performance outcomes, and underlying mechanisms remain inadequately explored. Herein, we propose a controllable nitrogen‐vacancy engineering strategy for GCN that induces interlayer contraction, thereby promoting interfacial charge transport. The tailored nitrogen vacancies broaden optical absorption, enhance interlayer electronic coupling, suppress charge carrier recombination, and lower the out‐of‐plane charge‐transfer barrier. Consequently, the optimized photocatalyst exhibits a ∼ 23‐fold enhancement in the hydrogen evolution rate compared to pristine GCN, while maintaining excellent stability over 50 h of continuous illumination. This work establishes a viable regulation strategy of nitrogen vacancies for advancing g‐C_3_N_4_‐based photocatalysts toward large‐scale solar hydrogen production.

## Graphical abstract

By integrating nitrogen‐vacancy engineering with interfacial electronic optimization, this study achieves a marked enhancement in the hydrogen evolution performance of g‐C_3_N_4_‐based photocatalysts, providing a new design paradigm for photocatalytic water splitting.

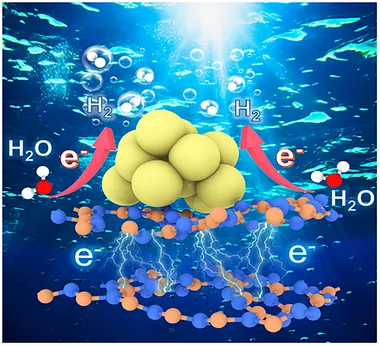

## 1 Introduction

Photocatalytic water splitting represents a highly attractive strategy for converting abundant solar energy into storable hydrogen fuel via semiconductor‐mediated redox reactions [1, 2, 3]. Among various photocatalysts, polymeric graphitic carbon nitride (g‐C_3_N_4_, GCN) has garnered considerable research interest due to its metal‐free nature, facile synthesis from abundant nitrogen‐rich precursors, and notable chemical and thermal stability [4, 5]. However, its photocatalytic efficiency is constrained by several inherent limitations: the relative and weak van der Waals interactions and large interlayer spacing impede interlayer charge transport [6, 7], also, its optical absorption is primarily restricted to the blue region of the visible spectrum. Furthermore, the material's highly symmetric structure and strong in‐plane bonding promote the rapid recombination of photogenerated electron–hole pairs, further limiting its photocatalytic performance [8, 9].

Over the last few decades, various strategies have been developed to enhance the photocatalytic performance of GCN [9, 10]. Structural modifications, such as reducing layer thickness or constructing porous architectures, can shorten the charge diffusion paths and increase the specific surface area [11, 12]. Tuning the electronic structure via doping or defect engineering is particularly effective for improving light absorption. For instance, introducing nitrogen vacancies creates mid‐gap states that extend the optical absorption edge into the visible region and accelerate charge separation [13, 14, 15]. Non‐metal doping can generate unpaired electrons, providing additional active sites for charge‐transfer [16, 17, 18]. Furthermore, interlayer engineering, such as intercalation of alkali metal cations [19, 20, 21] or direct control of the interlayer distance [22, 23, 24], can assist the formation of vertical charge‐transfer channels, promote interlayer charge migration, and suppress charge recombination. Collectively, these strategies typically increase the specific surface area, porosity, and light harvesting capability, contributing to improved photocatalytic activity.

Nevertheless, the controlled introduction of nitrogen vacancies and the precise understanding of their structure‐performance relationship remain significant challenges in GCN‐based photocatalysts [5, 19, 25]. Recent studies have elucidated the origin of the improved photocatalytic hydrogen evolution performance of defect‐modified GCN through morphology regulation, exfoliation or porous‐structure construction [26, 27]. However, compared with those approaches, the direct regulation of the concentration of nitrogen‐vacancies and the correlation between vacancy‐induced interlayer structural modulation and charge‐transport behavior remain less fully clarified [28, 29]. Moreover, in many reported nitrogen‐defective GCN systems, nitrogen vacancies are usually introduced through high‐temperature annealing, plasma treatment, chemical etching, or other post‐synthetic processes [15, 25, 27]. However, most reported approaches still rely on high‐temperature treatments or broadly non‐selective processes, which typically yield an uncontrolled and heterogeneous distribution of nitrogen vacancies [22, 30]. This variability obscures clear structure‐property relationships and complicates the correlation between vacant characteristics and photocatalytic performance [31, 32, 33]. Furthermore, the fundamental connections among vacancy density, modified electronic structure, and photocatalytic performance are not fully elucidated [34, 35]. Therefore, the rational design of high‐performance GCN photocatalysts demands both precise defect control strategies and a deeper mechanistic understanding of their electronic properties.

Herein, we report a controllable synthesis strategy to engineer nitrogen vacancies in GCN by modulating the glycine precursor ratio from 1 to 20 wt.% (denoted x% gly‐GCN, *x* = 1, 2, 5, 10, 20). Elemental analysis and powder X‐ray diffraction (PXRD) characterizations confirm the role of glycine in regulating the nitrogen vacancy concentration. The introduced vacancies correlate with a measurable contraction of the interlayer spacing (by 0.1%–0.6%), indicating a direct link between defect density and structural compression. Band structure analysis further reveals that the negatively shifted conduction band of gly‐GCN strengthens the reducing power of photogenerated electrons, which is beneficial for boosting the photocatalytic hydrogen evolution efficiency. Accordingly, 10% gly‐GCN achieves a hydrogen evolution rate ∼ 23 times higher than that of reference GCN, alongside excellent stability during prolonged operation. Spectroscopic, photoelectrochemical (PEC), and computational analyses confirm that the introduced nitrogen vacancies, together with interlayer contraction, promote charge separation, accelerate interlayer electron transport, and suppress charge recombination. This work elucidates the structure‐performance relationship in vacancy‐engineered GCN, providing a clear design principle for advanced photocatalysts.

## 2 Results and Discussion

### 2.1 Design Principle and Structural Characterizations of gly‐GCN

As shown in Figure 1a, the nitrogen vacancy concentration in the resulting carbon nitride was regulated by adjusting the precursor ratio of melamine to glycine (1, 2, 5, 10, and 20 wt.%), yielding a series of powder samples denoted as x% gly‐GCN (*x* = 1, 2, 5, 10, and 20). The thermal decomposition of glycine releases ammonia (NH_3_) and hydrogen cyanide (HCN) in situ [36]. These gaseous species create a reactive atmosphere that plays a key role in the structural evolution of GCN (Figure S1). During the high‐temperature thermal pyrolysis, the in situ released NH_3_ can partially dissociate into highly reactive radicals, such as ·H and ·NH_2_ [37, 38]. Under thermally activated conditions, these highly active species preferentially attack the thermodynamically unstable sites of GCN, including the amorphous carbon domains and the weakly bound bridging nitrogen sites at the edges of the heptazine frameworks [38]. Furthermore, this in situ chemical etching process can volatilize these less‐stable carbon‐ and nitrogen‐containing components into gaseous species, thereby introducing tunable nitrogen vacancies [39]. The introduction of glycine induced a distinct color change from pale‐yellow (reference GCN) to orange, with the color deepening as the glycine content increased (Figure S2). Scanning electron microscopy (SEM) images show that the overall morphology of the gly‐GCN series remained similar to that of GCN (Figure S3). Transmission electron microscopy (TEM) analysis revealed a clear structural difference: 10% gly‐GCN displayed a more fragmented and discontinuous nanosheet morphology compared to the continuous sheets of reference GCN (Figure 1b,c and Figures S4 and S5). Energy‐Dispersive X‐ray (EDX) elemental mapping confirms that both 10% gly‐GCN and GCN exhibit a uniform distribution of C and N species (Figure 1d and Figure S6).

**FIGURE 1:**
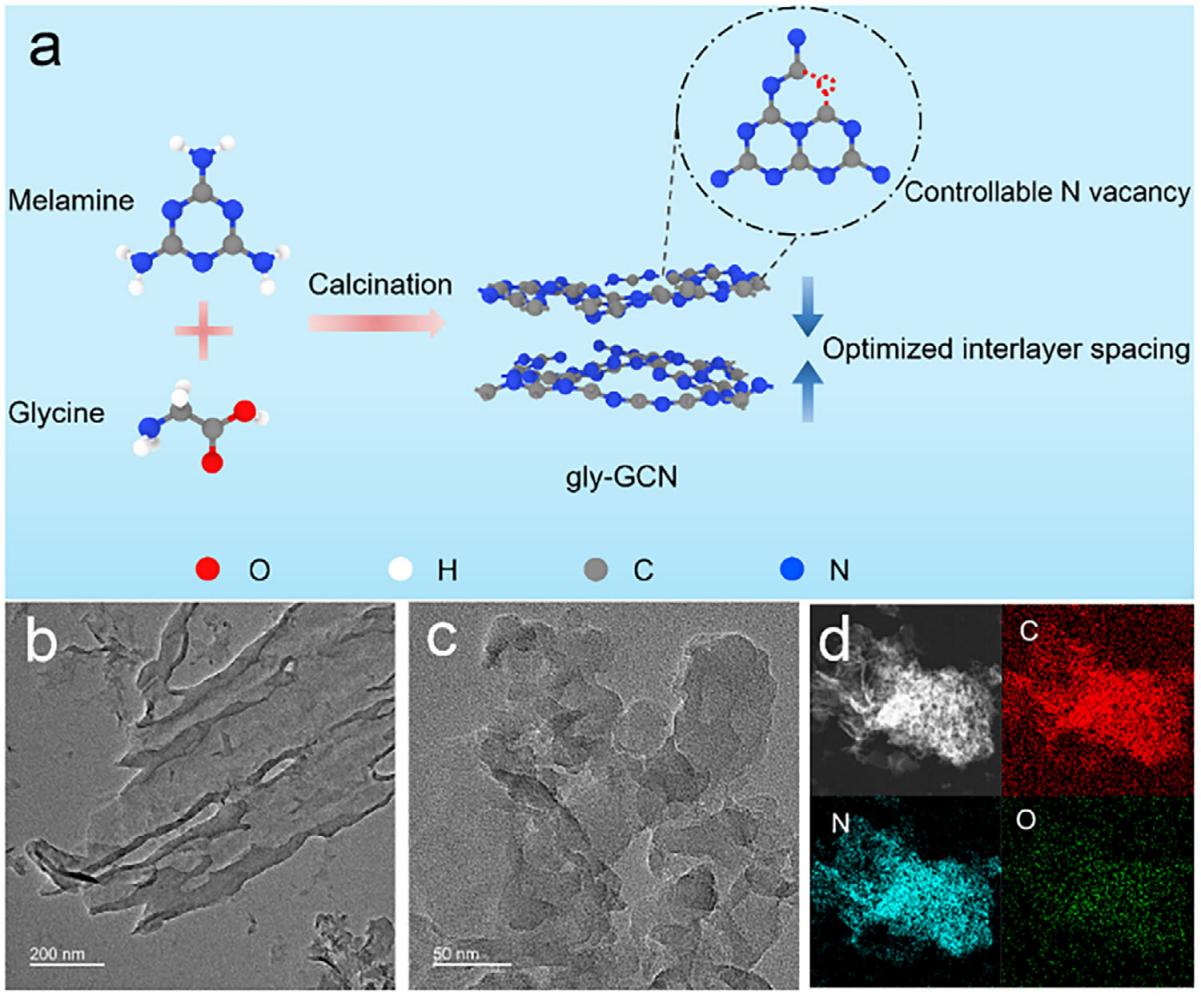
(a) Scheme of gly‐GCN synthesis. (b, c) TEM and HRTEM images of 10% gly‐GCN. (d) EDX elemental mapping of 10% gly‐GCN.

PXRD patterns of gly‐GCN and GCN (Figure S7) exhibit two characteristic diffraction peaks near 13° and 27°, assigned to the (100) and (002) planes of GCN, respectively [40]. The (100) peak position remains identical with increasing glycine content (Figure S8), while the (002) peak position gradually shifts from 27.49° to 27.64° as the glycine content increases (Figure S9). Since the (002) diffraction peak corresponds to the *π*–*π* stacking distance between GCN layers, this shift indicates a contraction of the interlayer spacing [23, 41]. As plotted in Figure 2a, the angular shift (Δθ, red line) increases from 0.03° to 0.18° as the glycine content increases from 1 to 20 wt.%, corresponding to a controlled reduction in the interlayer spacing (d_x_/d_0_, blue line), which decreases from 99.9% to 99.4% (a contraction of 0.1%–0.6%). In addition to the peak shift, the width of the (002) diffraction peak also changes after glycine modification. Compared with reference GCN, the gly‐GCN samples exhibit broader (002) reflections, indicating a decrease in the long‐range stacking order and crystallinity of the carbon nitride framework (Figure S9). This peak broadening can be associated with the introduction of nitrogen vacancies and local structural distortion during glycine‐assisted thermal polymerization [15, 42]. Therefore, glycine modification induces not only interlayer contraction but also a moderate change in stacking order, which may further influence charge transport and photocatalytic behavior. X‐ray photoelectron spectroscopy (XPS) analysis (Figure 2b and Figures S10 and S11) reveals collective shifts of the C 1s and N 1s peaks toward lower binding energies with increasing glycine content in GCN. Specifically, the C 1s component assigned to N─C═N shifts from 288.4 eV in GCN to 288.1 eV in 10% gly‐GCN (Figure 2b), while the N 1s component assigned to C═N─C slightly shifts from 398.6 eV in GCN to 398.5 eV in 10% gly‐GCN (Figure S11). In contrast, the O 1s spectra exhibit similar peak positions and profiles among the samples (Figure S12), suggesting that no dominant new oxygen‐related chemical environment is formed after glycine modification. Such collective shifts of the C 1s and N 1s peaks toward lower binding energy indicate an increased electron density localized on the sp^2^‐hybridized C and N atoms within the heptazine framework. This behavior can be rationalized by the formation of nitrogen vacancies: upon glycine incorporation, a fraction of the electronegative N atoms in GCN is removed or substituted, which reduces the local electrostatic potential and allows neighboring C and N atoms to accumulate more electron density. The higher electron density leads to more effective screening of the nuclear charge and therefore shifts the corresponding binding energies to lower values. Meanwhile, the nearly unchanged O 1s spectra suggest that the observed binding‐energy variation mainly originates from charge redistribution within the C─N framework rather than from the formation of new dominant oxygen‐containing species [43, 44]. In addition, the area ratio of the (C─C/C═C) peak at ∼284.8 eV to the (N─C═N) peak increases from ∼1:2.5 in GCN to ∼1:1.5 in 10% gly‐GCN (Figure 2b), indicating a progressive rise in sp^2^‐hybridized carbon domains that indirectly confirms an increase in nitrogen vacancy concentration.

**FIGURE 2:**
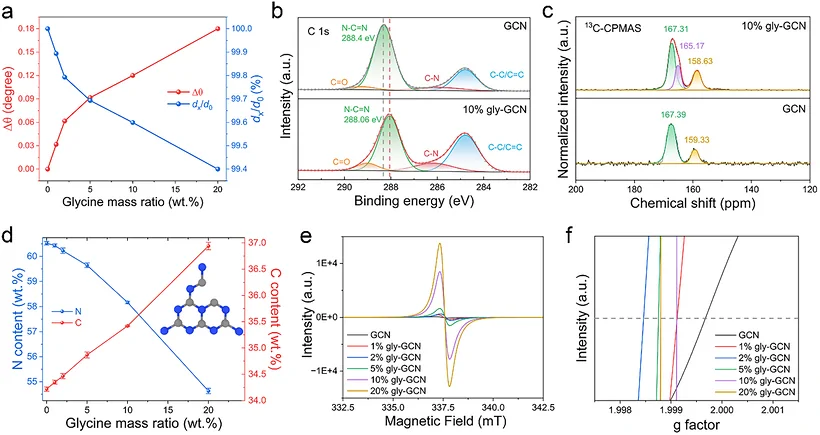
(a) Relationship between the glycine content and the shift of the (002) peak (Δθ, red line), as well as between the glycine content and the ratio of the tuned interlayer spacing d_x_ (*x* = 1, 2, 5, 10, 20 wt.%) to the interlayer spacing d_0_ of the reference GCN (d_x_/d_0_, blue line). (b) High‐resolution C 1s XPS of the different samples. (c) ^13^C CPMAS solid‐state NMR spectra of GCN and 10% gly‐GCN. (d) Relationship between the glycine content and the N (blue line) and C contents (red line) derived from results of C/H/N/S/O elemental analysis. The inset illustrates a nitrogen vacancy in the heptazine network. (e) EPR spectra of the different samples and (f) enlarged EPR spectra with g factors.

Solid‐state nuclear magnetic resonance (NMR) spectroscopy provides further evidence of the evolving electronic structure in gly‐GCN. In the ^13^C NMR spectra (Figure 2c), the resonance corresponding to C═N shifts from 159.3 ppm in GCN to 158.6 ppm in 10% gly‐GCN. Similarly, the two characteristic signals in the ^15^N NMR spectra shift downward from ‐244.3/‐265.5 ppm to ‐248.4/‐268.9 ppm (Figure S13). These coordinated shifts indicate enhanced nuclear shielding and increased local electron density, which is consistent with strengthened π‐conjugation due to the presence of nitrogen vacancies [45, 46]. An additional ^13^C component near 165 ppm suggests a subtle splitting of heptazine/triazine ring environments, reflecting a more electron‐rich framework with altered local bonding compared to reference GCN [47, 48]. Fourier transform infrared (FT‐IR) spectra (Figure S14) confirm that all gly‐GCN samples retain the characteristic vibrational modes of reference GCN. Notably, in the triazine region (Figure S15), the gly‐GCN series exhibits a new ring‐breathing mode at 875 cm^−1^, while the absorption band at 889 cm^−1^ is slightly redshifted to ∼887 cm^−1^. These spectral perturbations indicate a modified heptazine chemical environment, likely arising from vacancy formation during the thermal condensation of glycine with melamine [49].

Additionally, C/H/N/S/O elemental analysis reveals a close correlation between the nitrogenvacancy concentration and the glycine content in the precursor. As shown in Table S1, the relative nitrogen content gradually decreases from 60.44 ± 0.06 wt.% in 1% gly‐GCN to 58.17 ± 0.05 wt.% in 10% gly‐GCN, indicating the formation of nitrogen vacancies [40, 50]. This trend is mirrored in the nearly linear decrease of the N/C atomic ratio with increasing glycine content (Figure 2d and Table S2), which is consistent with a controlled introduction of nitrogen vacancies that subtly perturb the heptazine network (inset of Figure 2d) and its π stacking [40, 51]. Besides the decrease in nitrogen content, the oxygen content obtained from elemental analysis also changes after glycine modification. However, the O 1s XP spectra of GCN and gly‐GCN (Figure S12) exhibit similar peak positions and profiles, without the appearance of new dominant O‐related components, suggesting that substantial lattice oxygen incorporation or oxygen‐defect formation is not favorable. Therefore, this variation of oxygen content may be associated with surface‐adsorbed H_2_O/O_2_ species, residual oxygen‐containing groups formed during glycine‐assisted thermal polymerization, or slight surface oxidation during sample handling [14, 52]. Such oxygen‐related surface species may improve the hydrophilicity of GCN and could play a secondary role in modifying the surface properties [52, 53]. N_2_ adsorption–desorption isotherms (Figure S16) reveal that all gly‐GCN samples exhibit larger Brunauer–Emmett–Teller (BET) specific surface areas than reference GCN, while the average pore‐size distributions are broadly similar for all samples (inset of Figure S16). Notably, the pore volumes of the gly‐GCN series are significantly higher than that of reference GCN (Table S3), which can be ascribed to the gas‐evolution process during glycine decomposition. As discussed above, glycine thermally releases NH_3_ and HCN, creating a dynamic nitrogen‐rich atmosphere that selectively removes loosely bound or amorphous carbon domains from the heptazine framework, thereby enlarging the specific surface area and pore volume. Consequently, the resulting gly‐GCN materials provide more exposed active sites and improved mass transport for photocatalytic reactions.

Electron paramagnetic resonance (EPR) spectroscopy provides further evidence of defect generation. The signal intensity increases systematically with glycine content (Figure 2e and Figure S17), implicating a higher concentration of unpaired electrons (spin defects) in gly‐GCN. A slight decrease in the g value from 2.000 (reference GCN) to 1.998 (10% gly‐GCN) suggests enhanced π‐electron delocalization within the modified framework (Figure 2f) [54, 55]. In summary, nitrogen‐vacancy engineering concurrently tailors the composition, porosity, and electronic structure of GCN. This multi‐faceted modification increases the density of charge trapping sites, promotes electron delocalization, and enhances surface accessibility, collectively benefiting charge separation, transport, and interfacial reaction kinetics.

### 2.2 Band Structure Analysis and Photocatalytic Hydrogen Evolution Performance

To gain insight into the influence of nitrogen‐vacancy engineering on photocatalytic hydrogen evolution, we investigated the light‐absorption properties and band structure of the gly‐GCN series. UV–vis spectra (Figure 3a) showed a significant enhancement in the visible region (430–700 nm) with increasing glycine content, which should promote the generation of charge carriers under illumination. Corresponding Tauc plot analysis (Figure S18) revealed that the optical band gaps of the gly‐GCN samples are smaller than that of the reference GCN and gradually decrease with increasing glycine content. Ultraviolet photoelectron spectroscopy (UPS) demonstrates that the work function increases from ∼4.45 eV for GCN to ∼4.72 eV for 10% gly‐GCN (Figure 3b), indicating a downward shift of the Fermi level. This shift raises the electron emission barrier, helping to suppress charge recombination. XPS valence‐band (XPS‐VB) measurements show that the valence‐band maximum (VBM) moves close to the Fermi level in gly‐GCN (Figure S19), indicating facilitated hole extraction. The band‐edge positions in Figure 3d were constructed based on Mott–Schottky analysis and UV–vis results. As shown in Figure 3c and Figure S20, both GCN and 10% gly‐GCN exhibit positive slopes in the Mott–Schottky plots, confirming their n‐type semiconductor characteristics. For n‐type GCN‐based semiconductors, the conduction‐band potential is generally approximated to be ca. 0.1 V more negative than the flat‐band potential obtained by Mott–Schottky analysis [52]. The corresponding VB potentials were then calculated by combining the estimated conduction‐band (CB) potential with the optical bandgaps derived from Tauc plots. The band structure results in Figure 3d show that 10% gly‐GCN possesses a more negative CB potential than reference GCN, suggesting an enhanced driving force for photogenerated electron transfer and proton reduction under photocatalytic conditions.

**FIGURE 3:**
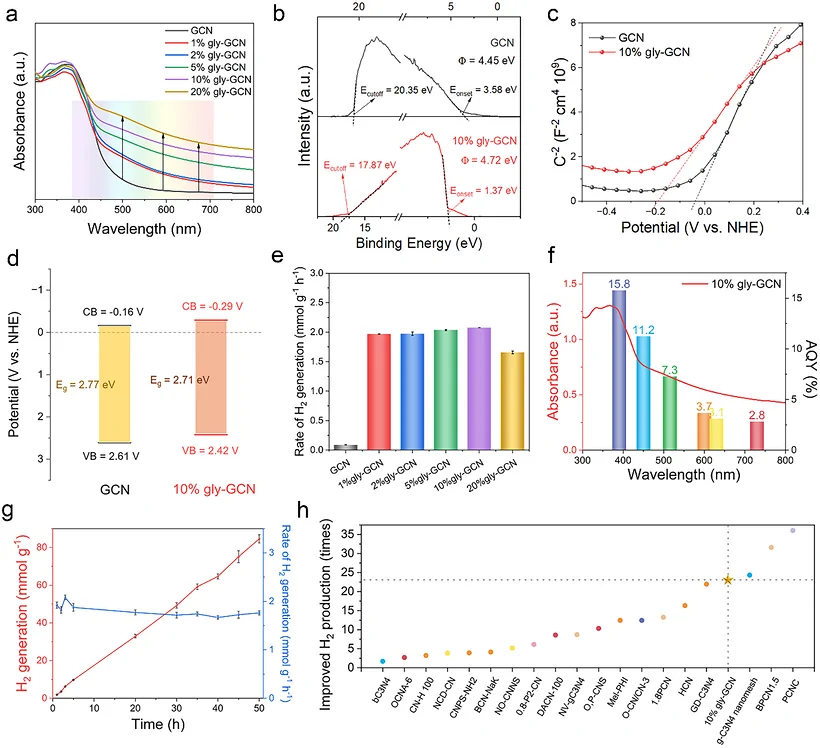
(a) UV–vis absorption spectra of GCN and a series of gly‐GCN samples. (b) UP spectra of GCN and 10% gly‐GCN under a bias of ‐5 V. (c) Mott–Schottky plots of GCN and 10% gly‐GCN. (d) Schematic illustration of band structures. (e) Photocatalytic hydrogen evolution rates. (f) UV–vis spectrum (red line) and the corresponding AQY values (bars) at different wavelengths. (g) 50 h photocatalytic hydrogen evolution stability test of 10% gly‐GCN. (h) Comparison of the photocatalytic hydrogen evolution performance observed in this work with recently reported related systems.

To evaluate the photocatalytic hydrogen evolution performance, the gly‐GCN series was dispersed in a 10 vol.% TEOA solution, and 3 wt.% Pt was photodeposited in situ on each sample as a cocatalyst following the procedure detailed in the Experimental Section. To optimize the catalyst loading, we varied the catalyst concentration and quantified both the H_2_ amount and the corresponding evolution rate (Figure S21). The H_2_ yield increases nearly linearly from 0.1 to 0.6 g L^−1^ but tends to plateau above 0.7 g L^−1^. The highest rate is obtained at 0.5 g L^−1^, which was therefore used for all comparative activity measurements unless otherwise stated. All gly‐GCN catalysts exhibit substantially enhanced hydrogen evolution rates (Figure 3e) in the following order: 10% gly‐GCN (2.07 mmol g^−1^ h^−1^) ≈ 5% gly‐GCN (2.04 mmol g^−1^ h^−1^) > 2% gly‐GCN (1.98 mmol g^−1^ h^−1^) > 1% gly‐GCN (1.91 mmol g^−1^ h^−1^) > 20% gly‐GCN (1.66 mmol g^−1^ h^−1^) ≫ reference GCN (0.09 mmol g^−1^ h^−1^). Thus, the 10% gly‐GCN sample is about 23 times more active than reference GCN (Figure 3e). The slightly lower activity of 20% gly‐GCN compared to other gly‐GCN samples may be attributed to excessive denitrogenation of the heptazine network. Excessive removal of lattice N atoms partially disrupts the heptazine network and increases the fraction of deep defect states that behave as recombination centers rather than productive reaction sites, thereby decreasing the effective population of catalytically active sites. 10% gly‐GCN maintains a high hydrogen evolution efficiency over the entire visible‐light range (Figure S22), far exceeding the typical near‐ultraviolet operation window (300–400 nm) of GCN. The apparent quantum yield (AQY) measurements further demonstrate the enhanced visible‐light photocatalytic activity of the modified samples compared to GCN (Figure 3f and Figure S23). Specifically, the AQY of 10% gly‐GCN is determined as 15.8% at 390 nm, which is much higher than that of GCN (0.8% at 390 nm). Even at longer wavelengths, 10% gly‐GCN still maintained measurable AQY values, while GCN showed much lower photon‐to‐hydrogen conversion efficiency. Long‐term hydrogen evolution tests (Figure 3g, blue line) show that 10% gly‐GCN maintains nearly constant activity over 50 h, with a total performance loss of only ∼3.5% over the test period. Meanwhile, 10% gly‐GCN also exhibits excellent cycling stability, with its hydrogen evolution activity showing almost no loss over three successive 5 h photocatalytic runs (Figure S24). To further evaluate the structural stability of the catalysts, the Pt‐deposited 10% gly‐GCN sample (Pt/10% gly‐GCN) was collected after the long‐term photocatalytic hydrogen evolution reaction and subjected to post‐catalytic XRD and TEM analyses. As shown in Figure S25a, the diffraction peaks of GCN and 10% gly‐GCN remain identical with and without Pt deposition, with no obvious secondary phase formation, suggesting that the GCN framework remains stable during Pt photo‐deposition process. Meanwhile, the main (002) diffraction peaks of Pt/10% gly‐GCN are largely retained after long‐term reaction, although slight changes in peak intensity may arise from sample recovery or partial exfoliation during photocatalysis (Figure S25b). The TEM images of Pt/10% gly‐GCN after a 50 h photocatalytic test also show that the original nanosheet morphology is largely preserved, and no severe structural collapse is observed (Figure S26). Moreover, the Pt nanoparticles remain stable on the GCN matrix without obvious large‐scale aggregation. These post‐catalytic characterizations, together with the visible‐light‐driven hydrogen evolution during the long‐term test, demonstrate efficient photocatalytic hydrogen evolution and robust operational durability of 10% gly‐GCN. Overall, the performance of 10% gly‐GCN is highly competitive among state‐of‐the‐art modified GCN photocatalysts (Figure 3h and Table S4) [14, 18, 20, 31, 56, 57, 58, 59, 60, 61, 62, 63, 64, 65, 66, 67, 68, 69, 70].

### 2.3 PEC Evaluation and Electronic Properties of gly‐GCN

The role of nitrogen vacancies and interlayer contraction in enhancing interfacial charge‐transfer was further investigated using KPFM and a series of PEC measurements. Thin films of 10% gly‐GCN and pristine GCN were prepared on FTO substrates via electrophoretic deposition [71]. The surface photopotentials of gly‐GCN and reference GCN under dark and illuminated conditions were probed by KPFM (Figure 4a). Both gly‐GCN and reference GCN form ∼100 nm‐sized particulate coatings on the FTO surface with film thicknesses of 60–100 nm (Figures S27 and S28). Contact potential difference (CPD) analysis along the line‐scan region under illumination shows that gly‐GCN exhibits a surface potential change of ∼60 mV, significantly larger than the ∼15 mV shift observed for GCN, indicating superior charge separation (Figure 4b). PEC measurements were carried out in 0.5 M Na_2_SO_4_ (pH 7) under both dark and illuminated conditions. Linear sweep voltammetry showed that the gly‐GCN electrode has a photocurrent density of ∼35.3 µA cm^−2^ at 0 V vs RHE, surpassing that of the reference GCN electrode (∼21.1 µA cm^−2^) (Figure 4c). The photoconversion efficiency η of the gly‐GCN electrode reached ∼0.27% at 0.16 V vs RHE, nearly double the value for GCN (∼0.15% at 0.13 V vs RHE) (Figure 4d). Transient photocurrent responses (Figure 4e) reveal that gly‐GCN generates significantly higher and more reproducible photocurrents during repeated on/off cycles, confirming enhanced charge separation and carrier mobility. Furthermore, electrochemical impedance spectroscopy (EIS) characterization shows a smaller charge‐transfer resistance for gly‐GCN under both dark and illuminated conditions (Figure 4f). Collectively, these PEC results corroborate that nitrogen vacancy engineering, coupled with interlayer contraction, significantly enhances charge separation and interfacial charge‐transfer, explaining the superior photocatalytic hydrogen evolution performance of gly‐GCN.

**FIGURE 4:**
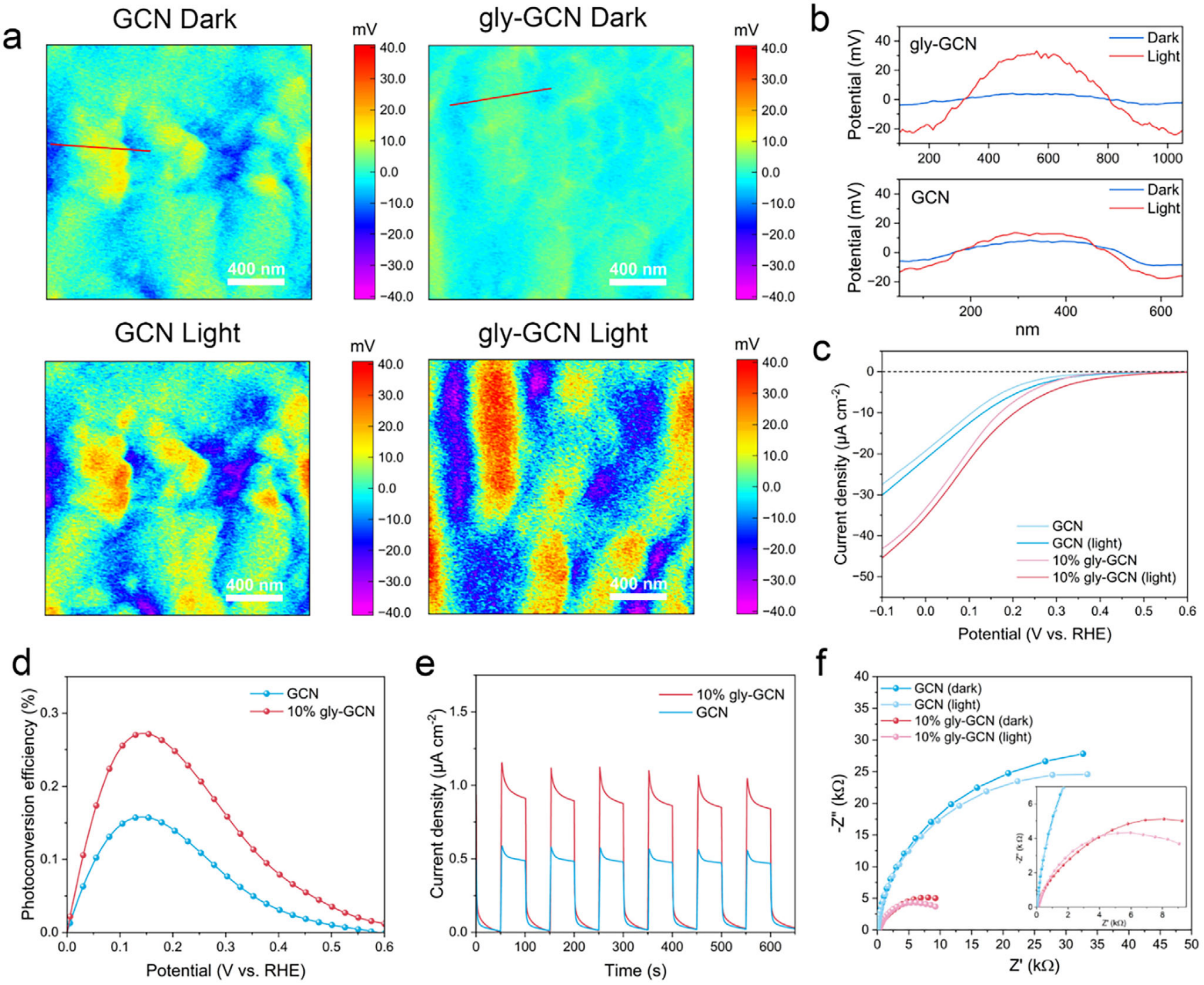
(a) KPFM maps of GCN and 10% gly‐GCN under dark and illuminated conditions. (b) Surface potential profiles along the red lines shown in (a). (c) LSV curves measured in the dark and under illumination. (d) Photoconversion efficiency curves. (e) Photocurrent responses. (f) EIS Nyquist plots recorded in the dark and under illumination, with the inset showing the locally enlarged Nyquist plots.

Photoluminescence (PL) and time‐resolved PL (TRPL) spectroscopy further elucidate charge carrier dynamics in gly‐GCN. The steady‐state PL intensities of the gly‐GCN powders decrease progressively with increasing glycine content (Figure 5a), indicating more effective suppression of radiative recombination. TRPL measurements (Figure 5c and Figure S29) reveal a shortening of the average lifetime in the gly‐GCN series, particularly through a pronounced reduction of the ∼10 ns component, consistent with accelerated charge‐transfer and suppressed electron–hole recombination. Together with the enhanced photocurrent response and the decreased charge‐transfer resistance, these results suggest that the dominant non‐radiative process arises from the rapid transfer of photogenerated carriers to interfacial catalytic sites rather than simple bulk non‐radiative recombination. Consequently, although the TRPL lifetimes become shorter, the improved interfacial charge separation and transfer enable more electrons to participate in the hydrogen evolution reaction, leading to a markedly enhanced photocatalytic hydrogen production activity of the gly‐GCN samples.

**FIGURE 5:**
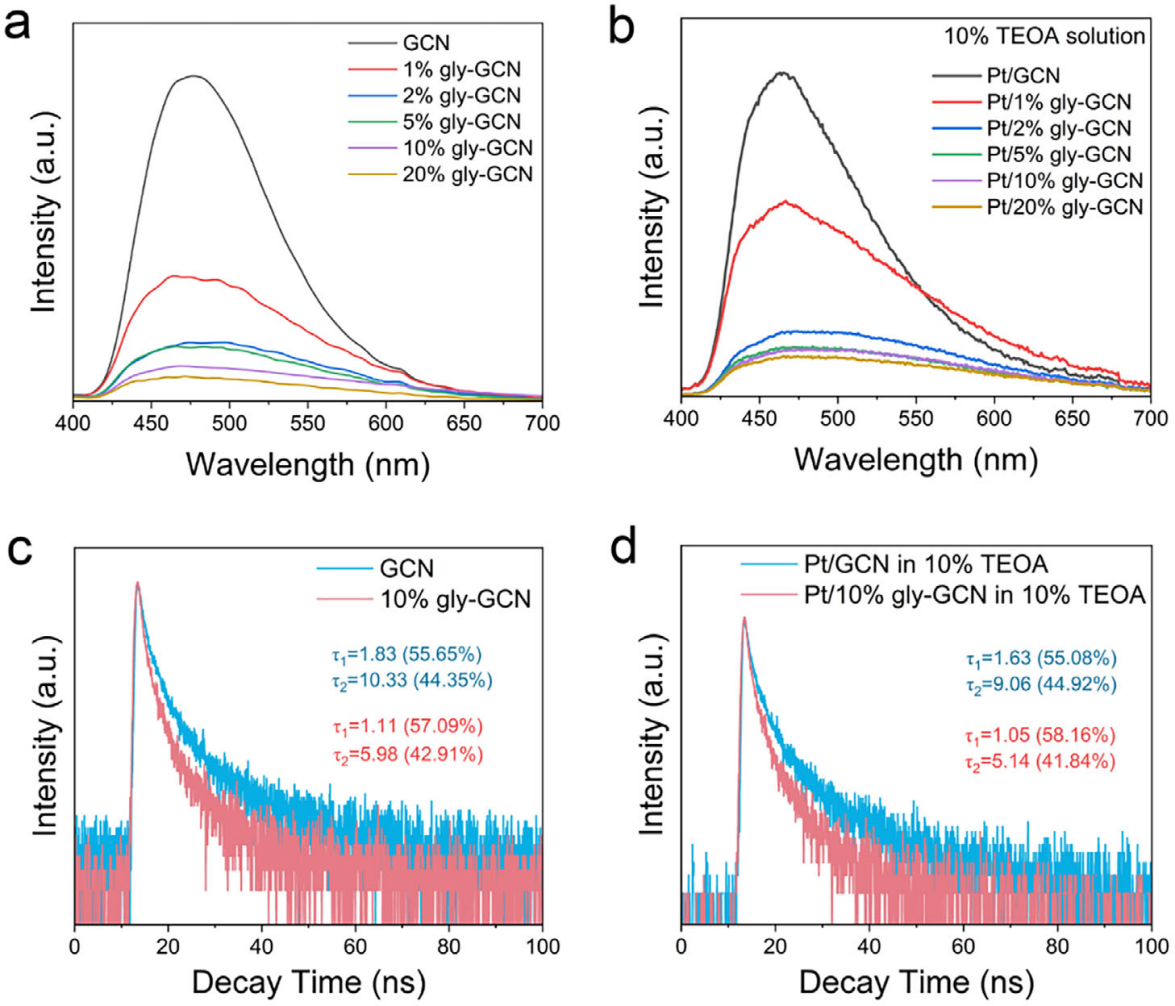
(a) PL spectrum, (c) TRPL decay curves and the corresponding fitted lifetime components of GCN and 10% gly‐GCN. (b) PL spectrum, (d) TRPL decay curves under photocatalytic conditions and the corresponding fitted lifetime components of Pt/GCN and Pt/10% gly‐GCN.

When measured in aqueous suspension under photocatalytic conditions, the PL quenching and lifetime shortening are even more pronounced for Pt‐loaded gly‐GCN (Figure 5b,d and Figure S30). The further reduced lifetimes confirm that photogenerated electrons are rapidly extracted from the gly‐GCN framework and transferred to the Pt cocatalysts. It is noteworthy that 20% gly‐GCN exhibits stronger visible‐light absorption and more pronounced TRPL decay behavior than 10% gly‐GCN, whereas its photocatalytic hydrogen evolution activity is slightly lower than 10% gly‐GCN. This indicates that photocatalytic performance is not solely determined by light absorption or carrier lifetime, but by the balance among photon harvesting, charge separation, charge transport, defect concentration, and surface reaction kinetics [15]. The stronger absorption of 20% gly‐GCN may originate partly from defect‐related sub‐bandgap states induced by the higher glycine content. Although these states broaden the visible‐light response, excessive defect formation can induce deep trap states and non‐radiative recombination centers [30, 50]. Similarly, a shorter TRPL lifetime may reflect accelerated charge‐transfer, but in highly defective samples it may also be associated with non‐radiative decay [72]. Therefore, the slightly lower hydrogen evolution activity of 20% gly‐GCN compared with 10% gly‐GCN can be attributed to excessive defect concentration and increased structural disorder, which partially offset the benefits of enhanced light absorption. Overall, these spectroscopic observations collectively demonstrate that 10% gly‐GCN provides an optimal balance between nitrogen‐vacancy engineering, charge separation, interfacial electron transfer, and preservation of sufficient structural order, resulting in optimal photocatalytic hydrogen evolution performance.

### 2.4 Computational Studies

To elucidate how nitrogen‐vacancy engineering affects the interlayer structure, electronic properties, and photocatalytic hydrogen evolution process of GCN, two Pt‐decorated bilayer GCN models were constructed: an intact GCN framework decorated with Pt particles, denoted as Pt/GCN, and a nitrogen‐vacancy‐containing model, denoted as Pt/N_v_‐GCN (Figure S31). Here, N_v_ represents a nitrogen vacancy. In the Pt/N_v_‐GCN model, a single nitrogen vacancy was introduced by removing a two‐coordinate bridging N atom (N_2_C site) from the heptazine network of the top GCN layer, and the Pt nanoparticle was placed directly at the defective site. The whole structure was then fully relaxed without imposing a reduced interlayer distance, allowing local structural optimization around the vacancy and at the interface.

After structural relaxation, the optimized interlayer spacing decreased from 0.388 nm for Pt/GCN to 0.342 nm for Pt/N_v_‐GCN, indicating that the introduction of nitrogen vacancies can lead to an interlayer contraction in the local GCN structure. Such a contraction originates from stronger interlayer interaction and enhanced electronic coupling between Pt and GCN. Consistently, the experimental PXRD results demonstrate that the (002) stacking reflection shifts from 2θ = 27.48° for Pt/GCN to 27.61° for Pt/10% gly‐GCN, corresponding to a decreased d_002_ spacing from 0.3242 to 0.3228 nm according to Bragg's law (Figure S25a). Although the absolute interlayer distances obtained from DFT and PXRD differ because the DFT model represents an idealized local structure, whereas PXRD provides an average stacking distance over the bulk sample, all the obtained results reveal the same trend upon introducing nitrogen vacancies.

The differential charge density maps (Figure 6a,b) reveal more pronounced electron accumulation around Pt and stronger charge‐transfer across the interface for Pt/N_v_‐GCN than in Pt/GCN. Bader charge analysis (Figure 6c,d) further confirms that the Pt atoms adjacent to the GCN surface gain more electron density. This tunes the catalytic properties of Pt toward enhanced hydrogen evolution activity by facilitating interfacial electron transfer and promoting more favorable adsorption of H^*^ on the Pt sites. Analysis of the projected density of states (PDOS) (Figure 6e) shows that, in Pt/N_v_‐GCN, the d‐band center of Pt shifts away from the Fermi level, weakening the adsorption of H^*^ and thereby facilitating hydrogen desorption [73]. Compared to Pt/GCN, the calculated activation barrier for H_2_O dissociation is reduced in Pt/N_v_‐GCN (Figure 6f), making the formation of H^*^ and OH^*^ more favorable. Consistently, the calculated hydrogen desorption energy on Pt/N_v_‐GCN is also lower, accelerating the overall hydrogen evolution reaction (Figure 6g). These theoretical results are in excellent agreement with the previous experimental observations. Therefore, the interlayer contraction induced by nitrogen vacancies enhances interfacial electron transfer, optimizes the electronic states of surface Pt atoms, and simultaneously promotes water dissociation and hydrogen desorption. Overall, theoretical and experimental results collectively demonstrate that nitrogen‐vacancy engineering in GCN effectively strengthens interfacial charge‐transfer, thereby significantly improving its photocatalytic hydrogen evolution performance.

**FIGURE 6:**
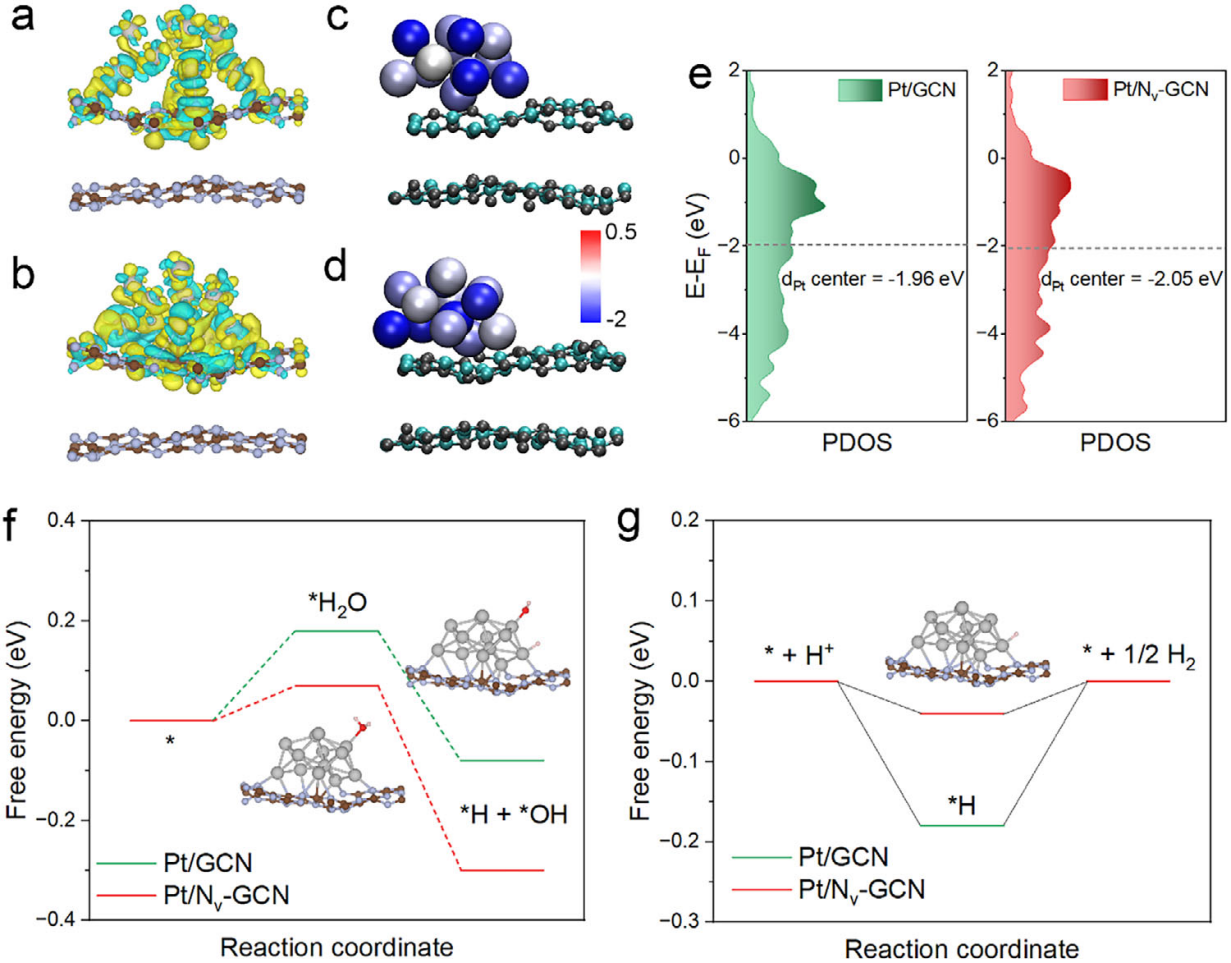
Differential charge density distributions of (a) Pt/GCN and (b) Pt/N_v_‐GCN, where the yellow and cyan regions represent electron accumulation and electron depletion, respectively. Light brown spheres represent carbon atoms, and bluish gray spheres represent nitrogen atoms. Bader charge maps of (c) Pt/GCN and (d) Pt/N_v_‐GCN; the top atom colors in (c, d) correspond to the calculated Bader charge values according to the inserted color scale (dark spheres: carbon atoms, cyan‐green spheres: nitrogen atoms). (e) PDOS of Pt 3d orbitals and the corresponding d‐band center positions. (f) Free energy diagram for water dissociation on the catalyst surface. (g) Free energy diagram for H adsorption/desorption on Pt sites.

## 3 Conclusions

This study demonstrates that the nitrogen‐vacancy concentration in GCN can conveniently and efficiently be regulated by the glycine content in the precursor materials, while simultaneously inducing a contraction of the interlayer spacing. Elemental analysis and PXRD characterizations show that the amount of nitrogen vacancies in gly‐GCN gradually increases with the amount of glycine in the starting materials. The relative change in the tuned interlayer spacing compared with reference GCN also shows a correlated decrease with increasing glycine content (degree of contraction adjustable within 0.1%–0.6%), which in turn indicates a clear correlation between the increase in nitrogen vacancies and the interlayer distance contraction. The resulting vacancy‐rich gly‐GCN exhibits enhanced visible‐light absorption, an optimized band structure with a downshifted conduction band, and markedly improved interfacial electron transfer. Thus, 10% gly‐GCN achieves a hydrogen evolution rate 23 times higher than that of GCN, while maintaining excellent stability during 50 h photocatalytic testing. Steady‐state and time‐resolved PL measurements indicate that the introduction of nitrogen vacancies facilitates charge‐transfer and suppresses electron‐hole recombination, and PL/TRPL measurements under photocatalytic conditions confirm that electrons can be rapidly transferred from gly‐GCN to Pt cocatalyst nanoparticles. Moreover, KPFM measurements, PEC evaluations, and theoretical calculations show that gly‐GCN with tunable nitrogen‐vacancy contents enhanced interfacial electron transfer, improved H^+^ adsorption–desorption kinetics, reduced the energy barrier for water dissociation, leading to synergistic promotion of hydrogen generation. In summary, this work shows that precursor‐controlled nitrogen‐vacancy engineering effectively enhances the photocatalytic hydrogen evolution performance of g‐C_3_N_4_, providing a general design principle for the rational optimization of g‐C_3_N_4_‐based photocatalysts.

## Funding

This work was supported by the University Research Priority Program (URPP) for Solar Light to Chemical Energy Conversion (LightChEC).

## Conflicts of Interest

The authors declare no conflict of interest.

## Supporting information




**Supporting File**: smll75195‐sup‐0001‐SuppMat.pdf.

## Data Availability

The data that support the findings of this study are available from the corresponding author upon reasonable request.
