# Supplementary material for: Engineering Nitrogen Vacancies in Polymeric Carbon Nitride to Promote Interfacial Electron Transfer for Visible‐Light Driven Hydrogen Evolution
Source: Small. 2026 Aug 21;22(58):e75195. doi: 10.1002/smll.75195 (PMC13676839; doi:10.1002/smll.75195)
Supplement: Supplementary file 1 [file SMLL-22-e75195-s001.pdf]

Supporting Information

Engineering Nitrogen Vacancies in Polymeric Carbon Nitride to Promote Interfacial Electron Transfer for Visible-Light Driven Hydrogen Evolution

Zeyi Zhang[a], Han Zhao[a], Lingshen Meng[a], Yu Wang[a], Fei Guo[b], Carlos A. Triana[a], Yonggui Zhao[a*], Jingguo Li[c*], Xiaoyuan Liu[d*], Greta R. Patzke[a*]

[a] Department of Chemistry, University of Zurich, Winterthurerstrasse 190, Zurich, 8057, Switzerland

[b] Department of Chemical Engineering, University College London, London, WC1E 7JE, United Kingdom

[c] State Key Laboratory of Advanced Environmental Technology, School of Environment, University of Science and Technology of China, Hefei, 230026, China

[d] Department of Chemistry and Key Laboratory for Preparation and Application of Ordered Structural Materials of Guangdong, Shantou University, Shantou, Guangdong, 515063, China

*Correspondence to: yonggui.zhao@chem.uzh.ch, lijg@ustc.edu.cn, xiaoyuanliu@stu.edu.cn, greta.patzke@chem.uzh.ch

Table of Contents

Chemicals.. 1

Materials Preparation 1

Materials Characterization 2

Photocatalysis Methods... 3

Photoelectrochemical Methods 5

DFT Calculations 6

Fig. S1. Schematic diagram of the formation of N-vacancies in GCN induced by products of glycine thermal decomposition. 7

Fig. S2. Photograph showing the color change of gly-GCN samples. 7

Fig. S3. SEM images of GCN and different gly-GCN samples. 8

Fig. S4. TEM images of 10% gly-GCN. 9

Fig. S5. TEM images of reference GCN. 10

Fig. S6. EDX elemental maps of reference GCN. 11

Fig. S7. XRD patterns of different GCN samples. 12

Fig. S8. XRD patterns of different samples in the 2θ range of 10-16°. 13

Fig. S9. XRD patterns of different samples in the 2θ range of 25-30°. 14

Fig. S10. High-resolution C 1s XP spectra of different GCN samples. 15

Fig. S11. High-resolution N 1s XP spectra of different GCN samples. 16

Fig. S12. High-resolution O 1s XP spectra of different GCN samples. 17

Fig. S13. ^{15}N CPMAS solid-state NMR spectra of GCN and 10% gly-GCN. 18

Fig. S14. FT-IR spectra of different GCN samples. 19

Fig. S15. Enlarged FT-IR spectra regions (zoom) of different GCN samples. 20

Fig. S16. N_2 adsorption-desorption isotherms and the corresponding pore size distribution. 21

Fig. S17. Enlarged EPR spectra (zoom) of different GCN samples. 22

Fig. S18. Tauc plots of different GCN samples. 23

Fig. S19. XP valence band spectra of the different GCN samples. 24

Fig. S20. Mott-Schottky plots measured at different frequencies. 25

Fig. S21. Optimization of 10% gly-GCN concentration for photocatalytic H_2 evolution. 26

Fig. S22. Photocatalytic H_2 evolution efficiency of the 10% gly-GCN sample at different wavelengths....27

Fig. S23. Photocatalytic H_2 evolution efficiency of reference GCN sample at different wavelengths and calculated AQY....28

Fig. S24. Cycling stability of 10% gly-GCN and GCN for photocatalytic H_2 evolution. 29

Fig. S25. PXRD patterns of Pt/GCN, Pt/10% gly-GCN, and Pt/10% gly-GCN after 50 h photocatalytic H_2 evolution....30

Fig. S26. TEM images of (a) Pt/10% gly-GCN and (b) Pt/10% gly-GCN after 50 h photocatalytic H_2 evolution. 31

Fig. S27. Three-dimensional topography maps of reference GCN sample obtained from AFM-KPFM measurements....32

Fig. S28. Three-dimensional topography maps of the 10% gly-GCN sample obtained from AFM-KPFM measurements....33

Fig. S29. TRPL spectra of different GCN powder samples....34

Fig. S30. TRPL spectra of different samples measured under photocatalytic hydrogen evolution conditions....35

Fig. S31. Calculated models of Pt/GCN and Pt/N_v-GCN....36

Table S1. C/H/N/S/O elemental analysis results of different GCN and gly-GCN samples....37

Table S2. Calculations of C/N, H/N, O/N and C/H/N/O ratio for different gly-GCN samples based on C/H/N/S/O elemental analysis results....38

Table S3. BET analysis of different GCN and gly-GCN samples....39

Table S4. Comparison of the photocatalytic H_2 evolution performance of the investigated GCN-based catalysts with recent representative studies....40

Chemicals

All chemicals were commercial and used without further purification: Melamine (2,4,6-Triamino-1,3,5-triazine, $C_3H_6N_6$; 99.0%, Sigma-Aldrich), Glycine (NH_2CH_2COOH; ACS reagent grade, ≥98.5%, powder, Sigma-Aldrich), Chloroplatinic acid hexahydrate ($H_2PtCl_6 \cdot 6H_2O$, ACS reagent, ≥37.50% Pt basis, Sigma-Aldrich), Ethanol (CH_3CH_2OH, ≥99.5%, ACS reagent, Sigma-Aldrich), Triethanolamine (TEOA, $(HOCH_2CH_2)_3N$, ≥99.0% (GC), Sigma-Aldrich), Fluorine doped tin oxide coated glass slide (SnO_2/F, FTO glass, L × W × thickness 20 mm × 10 mm × 3 mm, surface resistivity ~8 Ω/sq, Transmittance: 80-81.5% (visible), Sigma-Aldrich), Acetone (CH_3COCH_3, ACS reagent, ≥99.5%, Sigma-Aldrich), Iodine (I_2, ACS reagent, ≥99.8%, solid, Sigma-Aldrich), Argon (Ar 6.0, ≥99.9999%, Linde), and deionized (DI) water with the specific resistance of 18.25 $M\Omega \cdot cm^{-1}$ (obtained by reversed osmosis followed by ion-exchange and filtration).

Materials Preparation

Preparation of GCN

In a typical experiment, GCN was prepared by high-temperature thermal condensation of melamine. 2 g of melamine was placed in a covered alumina crucible and heated in a tubular furnace under Ar atmosphere to 550 °C at a ramp rate of 5 °C min^{-1}, followed by annealing at this temperature for 4 h. After cooling to room temperature, the resulting yellow bulk product was ground into a powder for 30 min, washed three times with DI water, and then vacuum-dried at 60 °C for 24 h to afford yellow GCN powder.

Preparation of gly-GCN

The gly-GCN series were obtained by thermal polycondensation of melamine and glycine mixed at different mass ratios. Taking 1% gly-GCN as an example, 1.98 g of melamine and 0.02 g of glycine (corresponding to 1 wt.% of the total precursor mass) were thoroughly ground together. The subsequent thermal polymerization and post-treatment steps were identical to those used for the synthesis of GCN. The resulting

powders, which exhibited different colors, were designated as 1%, 2%, 5%, 10%, and 20% gly-GCN according to the mass percentage of glycine in the precursor mixture.

Fabrication of GCN/FTO and gly-GCN/FTO

GCN or gly-GCN was deposited onto FTO glass substrates by electrophoretic deposition. Briefly, 1 cm × 2 cm FTO glass slides were sequentially immersed in acetone, ethanol, and deionized water, and ultrasonically cleaned in each solvent for 15 min. The deposition suspension was prepared by ultrasonically dispersing 4 mg of GCN or gly-GCN powder together with 4 mg of iodine in 50 mL of acetone. The FTO glass was used as the cathode and a Pt plate as the anode in a two-electrode configuration connected to a Multi EmStat 4 HR electrochemical workstation. Electrophoretic deposition was carried out at 5 V for 2 min to obtain GCN or gly-GCN films on the FTO substrates. The resulting GCN-coated FTO was then annealed at 450 °C for 1 h in a tubular furnace under Ar atmosphere, followed by natural cooling to room temperature, yielding GCN/FTO and gly-GCN/FTO photoelectrodes for Kelvin Probe Force Microscopy (KPFM) and PEC measurements.

Materials Characterization

Scanning Electron Microscopy (SEM) was carried out on a Zeiss SUPRA 50VP microscope. Transmission Electron Microscopy (TEM) images were obtained using an FEI Talos F200X, and Powder X-ray Diffraction (PXRD) patterns were recorded on a STOE STADI P diffractometer (transmission mode) equipped with a Cu K_α (1.54184 Å) radiation source (40 kV, 40 mA). X-ray Photoelectron Spectroscopy (XPS) measurements were performed on a Thermo Scientific ESCALAB 250X system. All XP spectra were calibrated by referencing the C-C component in the C 1s region to 284.8 eV. The same binding-energy correction was applied to all corresponding core-level spectra collected from the same sample. Fourier Transform Infrared Spectroscopy (FT-IR) spectra were recorded on a Bruker Vertex 70 FT-IR spectrometer.

N_2 adsorption-desorption isotherms were measured using a QuadraSorb four-channel

surface area and pore-structure analyzer. The total specific surface area was calculated using the Brunauer-Emmett-Teller (BET) method, and the pore size distribution curves were derived from the nonlocal density functional theory (NLDFT) model. C/H/N/S/O elemental analysis was performed on a HEKAtech EuroVector CHNS-O1 elemental analyzer. Room temperature Electron Paramagnetic Resonance (EPR) spectra were recorded on a Magnettech MS-5000 spectrometer. Solid state NMR spectra were acquired on a Bruker Avance III spectrometer using cross-polarization magic-angle spinning (CPMAS).

Kelvin Probe Force Microscopy (KPFM) measurements were performed under dark and illuminated conditions using an Asylum Research MFP-3D Origin AFM with BudgetSensors ElectriMulti75-G (Multi75E-G) conductive probes. A 300 W Xe lamp equipped with an AM 1.5G filter was used as the simulated solar-light source. The light intensity was calibrated to 100 mW cm^{-2} at the sample surface prior to the measurements. The measurements were conducted after the surface potential reached a stable state. UV-vis absorption spectra were recorded on a Shimadzu UV-3600 spectrometer. Photoluminescence (PL) and Time-Resolved PL (TRPL) spectra were obtained on an Edinburgh FLS1000 spectrometer with an excitation wavelength of 365 nm. Ultraviolet Photoelectron Spectroscopy (UPS) measurements were conducted on a Thermo ESCALAB 250Xi instrument. Gas chromatography (GC) was performed on an Agilent 7920A gas chromatograph. An Ossila LED solar simulator (AM 1.5) was used as simulated sunlight source, and photoelectrochemical (PEC) measurements were carried out on a Multi EmStat 4 HR electrochemical workstation.

Photocatalysis Methods

A closed-circulation quartz reactor with a quartz window on the top was used for photocatalytic measurements. The reactor was connected to a gas GC system in a closed gas-circulation line, and the air inside was thoroughly removed by N_2 purging. The reaction temperature was maintained at 25 °C throughout. For monochromatic apparent quantum yield (AQY) measurements, only the target wavelength was used, and the

effective irradiated area *S* was recorded.

The influence of catalyst concentration on photocatalytic hydrogen evolution was evaluated under the same reaction conditions. The catalyst concentration was adjusted by changing the catalyst mass while keeping the total solution volume (10 vol% TEOA aqueous solution) and all other parameters constant, including *in situ* photodeposition of 3 wt.% Pt. For each concentration (0.1-1.0 g L^{-1}), the evolved H_2 amounts were quantified by GC at regular intervals, and the hydrogen evolution rate was determined from the linear region of the H_2 evolution curve. The H_2 amount increases approximately linearly in the range of 0.1-0.6 g L^{-1}, whereas further increasing the concentration above 0.7 g L^{-1} gives diminishing increments. The maximum rate is achieved at 0.5 g L^{-1}, which therefore was used for subsequent activity comparisons. In a typical procedure, 50 mg of the photocatalyst was dispersed in 100 mL of 10 vol% TEOA solution and subjected to ultrasonic treatment for 15 min to ensure uniform dispersion. A 2.03 mL $H_2PtCl_6 \cdot 6H_2O$ solution (3.9 mmol L^{-1}) was added dropwise to achieve the desired Pt loading of 3 wt.% relative to the photocatalyst mass. The resulting suspension was stirred at 300 rpm and degassed by bubbling with high-purity N_2 for 30 min to remove dissolved oxygen and establish an inert atmosphere. Photodeposition was carried out under continuous stirring (300 rpm) and irradiation using a Ossila LED solar simulator (100 mW cm^{-2}) for 1 h. During irradiation, photogenerated electrons reduced Pt^{4+} ions to metallic Pt nanoparticles, which were selectively deposited on the surface of the semiconductor. After deposition, the Pt-loaded catalyst was collected by centrifugation, thoroughly washed with DI water and ethanol multiple times to remove residual ions and organic species, and then dried under vacuum at 60 °C for 6 h. The final products were designated as Pt/gly-GCN or Pt/GCN, respectively.

Photocatalytic H_2 evolution was carried out under continuous stirring at 300 rpm, and gas samples were taken at predetermined time intervals. The evolved H_2 was quantified using a gas chromatograph equipped with a thermal conductivity detector (GC-TCD),

with N_2 as the carrier gas. The H_2 amount was calculated using a pre-established calibration curve, where the peak area A and the H_2 quantity υ(H_2) follow a linear relationship:

$$A = 36.5\, v(\mathrm{H_2}) - 32.9\ (R^2 = 0.999)$$

Hydrogen evolution rates were normalized to the catalyst mass and expressed in μmol h^{-1} g^{-1}.

For stability tests, the reaction was conducted under identical conditions with continuous illumination for 50 h, and gas samples were collected at fixed intervals. The activity decay was evaluated from the time-dependent instantaneous H_2 evolution rate normalized to the catalyst mass.

The AQY was defined as:

$$\mathrm{AQY}(\%) = \frac{2\, n(\mathrm{H_2})\, N_\mathrm{A}\, hc}{P_\mathrm{in}\, S\, t/\lambda} \times 100$$

where $n(H_2)$ is the amount of H_2 produced (in moles), h is Planck's constant, c is the speed of light, N_A is Avogadro's constant, and λ is the wavelength of the incident light (in meters).

Photoelectrochemical Methods

All PEC measurements were carried out in a conventional three-electrode configuration. GCN/FTO or gly-GCN/FTO was used as the working electrode, with the effectively illuminated area defined as 1.0 cm^2 by masking the remaining region with heat-resistant waterproof tape. A 1 × 1 cm Pt foil served as the counter electrode, and a saturated calomel electrode (SCE) was used as the reference electrode. The electrolyte was 0.5 M Na_2SO_4 (pH 7.0), contained in a custom-made square fused-quartz electrochemical cell.

All measurements were performed at room temperature. To minimize the influence of dissolved oxygen, the electrolyte was purged with N_2 for 30 min prior to testing. The

light intensity was calibrated to 100 mW cm^{-2}, and dark current data were collected under steady-state conditions after switching off the light. All potentials were converted to the reversible hydrogen electrode (RHE) scale at 25 °C using:

$$E_{\mathrm{RHE}} = E_{\mathrm{SCE}} + 0.241\ \mathrm{V} + 0.0591 \times \mathrm{pH}$$

The photoconversion efficiency η was then calculated according to $\eta = (J_{Pmax} \times V_{Pmax})/P_{in}$, where J_{Pmax} and V_{Pmax} are the photocurrent density and photovoltage at the maximum power point, and P_{in} = 100 mW cm^{-2}.

DFT Calculations

All DFT calculations were performed using the CP2K package [1]. Core-valence interactions were described with Goedecker-Teter-Hutter (GTH) pseudopotentials, and the valence electronic states were expanded in Gaussian basis sets [2-5]. The DZVP-MOLOPT-SR-GTH basis set [2] was employed for all elements, together with a plane-wave cutoff of 400 Ry for the auxiliary density. Exchang-correlation effects were treated within the generalized gradient approximation of Perdew-Burke-Ernzerhof (PBE), combined with Grimme's D3 dispersion correction (DFT-D3) [6-8]. Self-consistent field (SCF) iterations were converged to an electronic energy tolerance of 1.0×10^{-6} Hartree. All structures were optimized using the Broyden-Fletcher-Goldfarb-Shanno (BFGS) algorithm until the maximum force was below 4.5×10^{-4} Hartree/Bohr. The Pt/GCN and Pt/GCN-nar models were constructed by modifying the C_3N_4 structure from the Materials Project database (mp-971684) [9]. In the Pt/GCN model, we constructed a defect-free bilayer GCN structure with an initial interlayer distance of 0.38 nm and placed a Pt nanoparticle on the heptazine network of the top GCN layer. In the Pt/GCN-nar model, a single nitrogen vacancy was introduced into the heptazine network, and the Pt nanoparticle was positioned directly at this defect site. Both structures were then fully relaxed to allow local reconstruction around the vacancy and at the Pt-GCN interface. These models are therefore intended to probe the local electronic and structural effects of an isolated N-vacancy-Pt site rather than to represent

a specific bulk vacancy concentration. For slab calculations, a vacuum region of 20 Å was added along the surface normal to eliminate spurious interactions between periodic images.

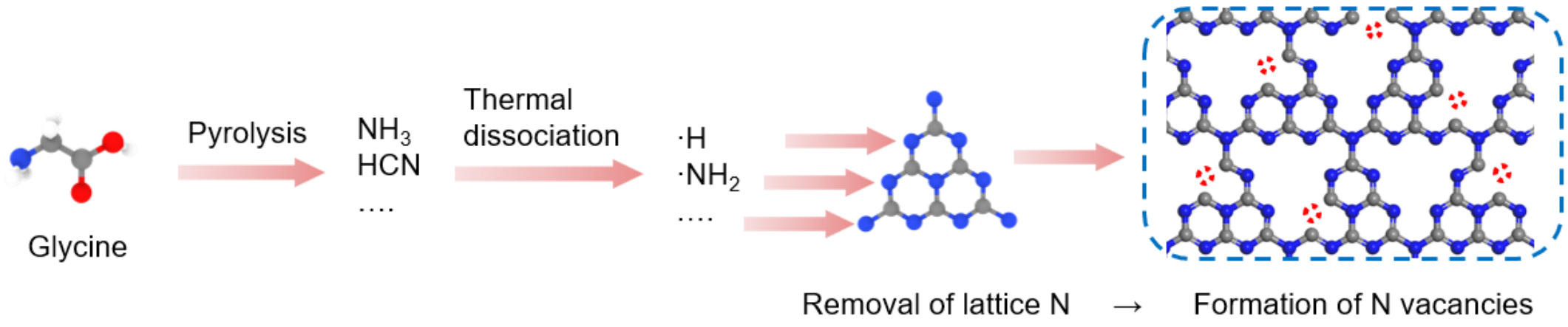

Fig. S1. Schematic diagram of the formation of N-vacancies in GCN induced by products of glycine thermal decomposition.

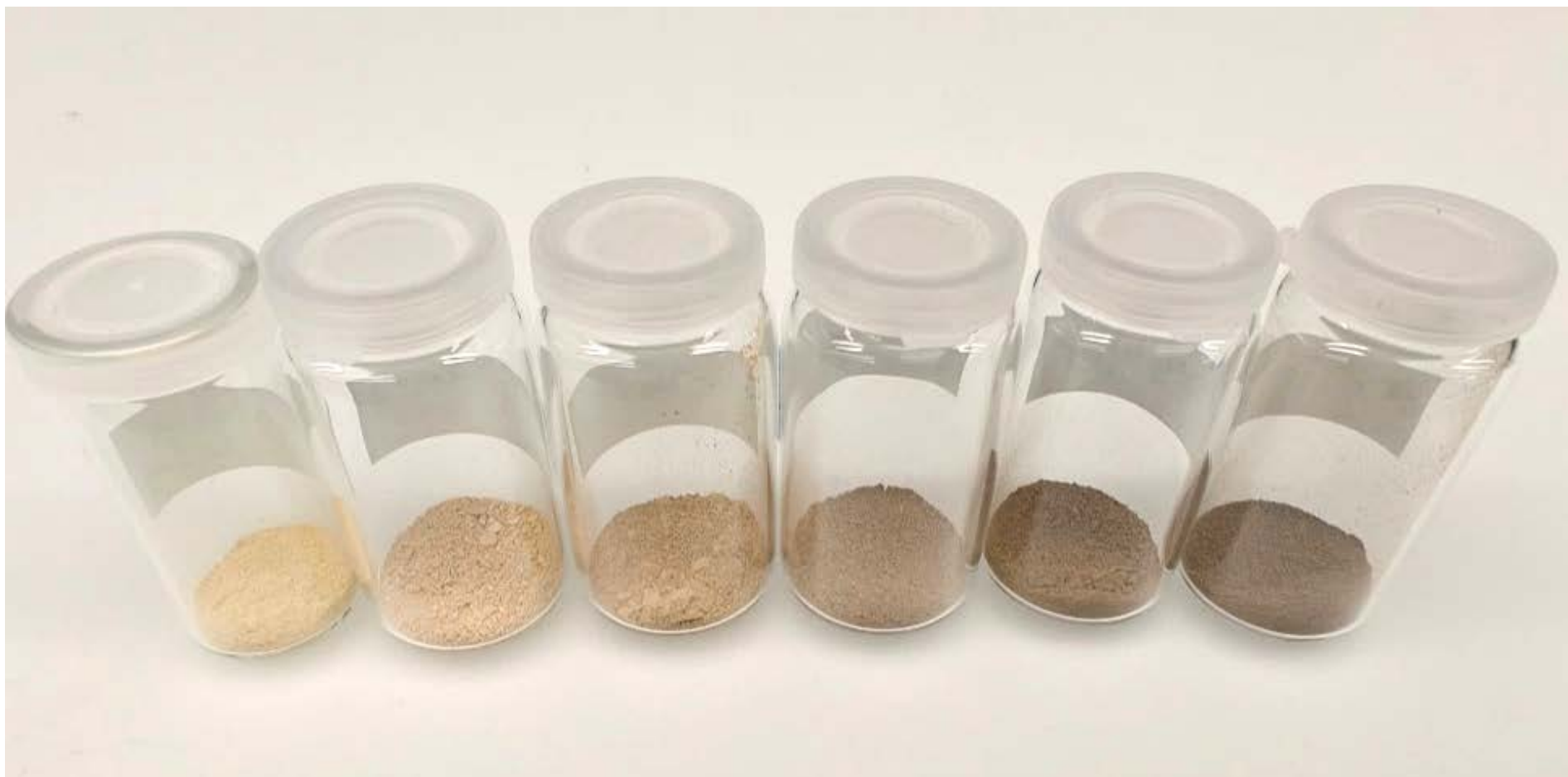

Fig. S2. Photograph showing the color change of gly-GCN samples. From left to right: GCN, 1%, 2%, 5%, 10%, and 20% gly-GCN.

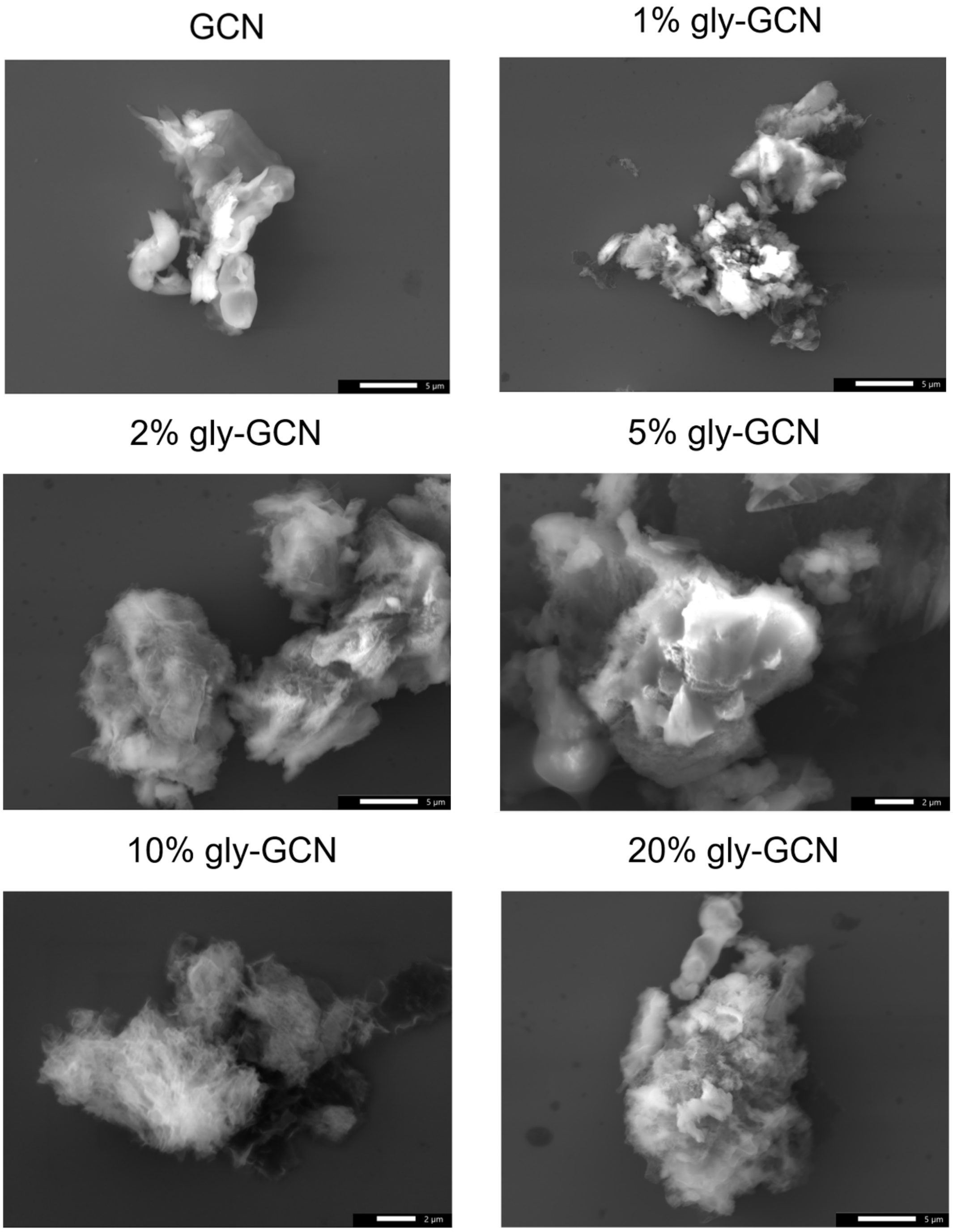

Fig. S3. SEM images of GCN and different gly-GCN samples.

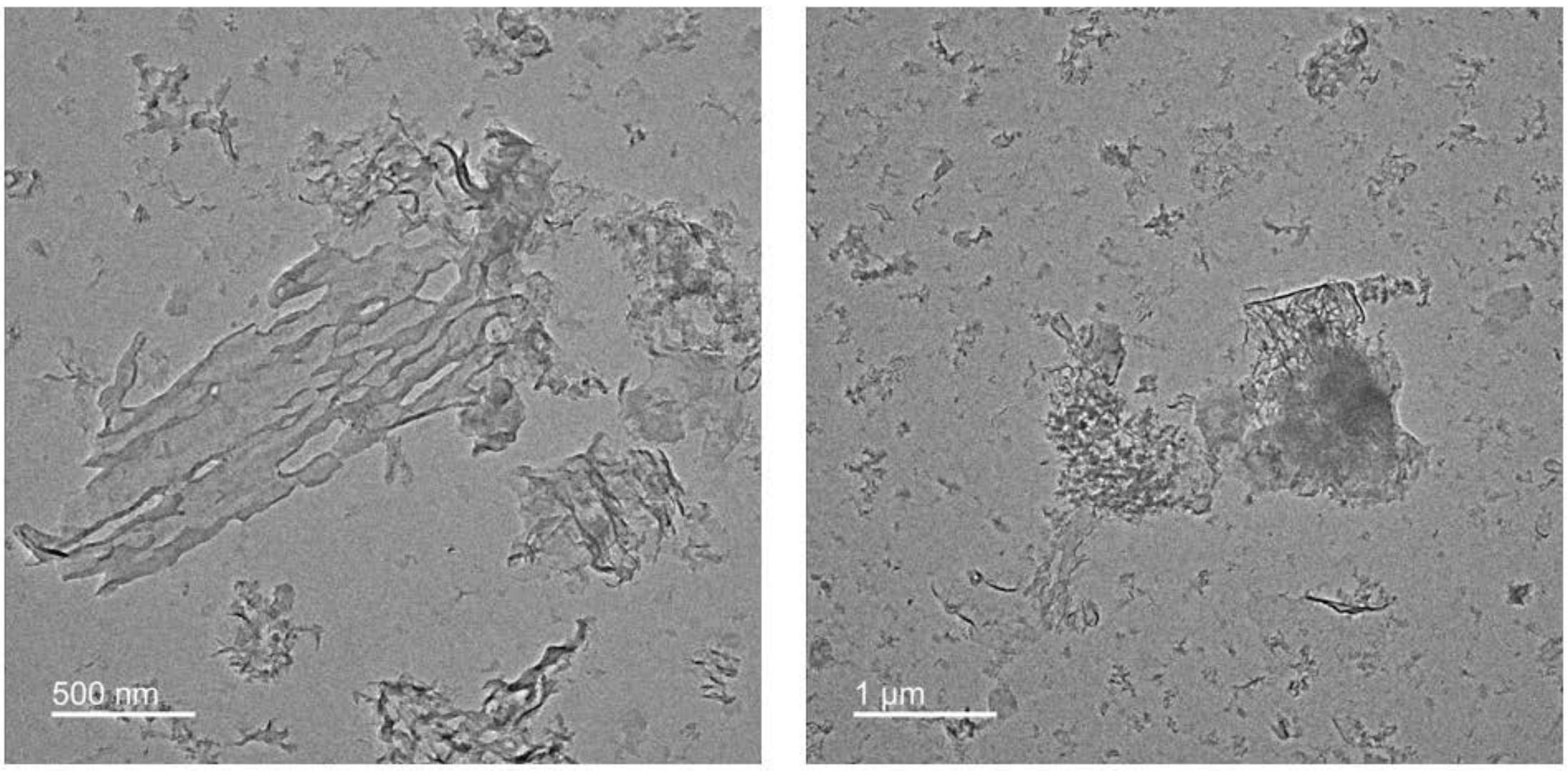

Fig. S4. TEM images of 10% gly-GCN.

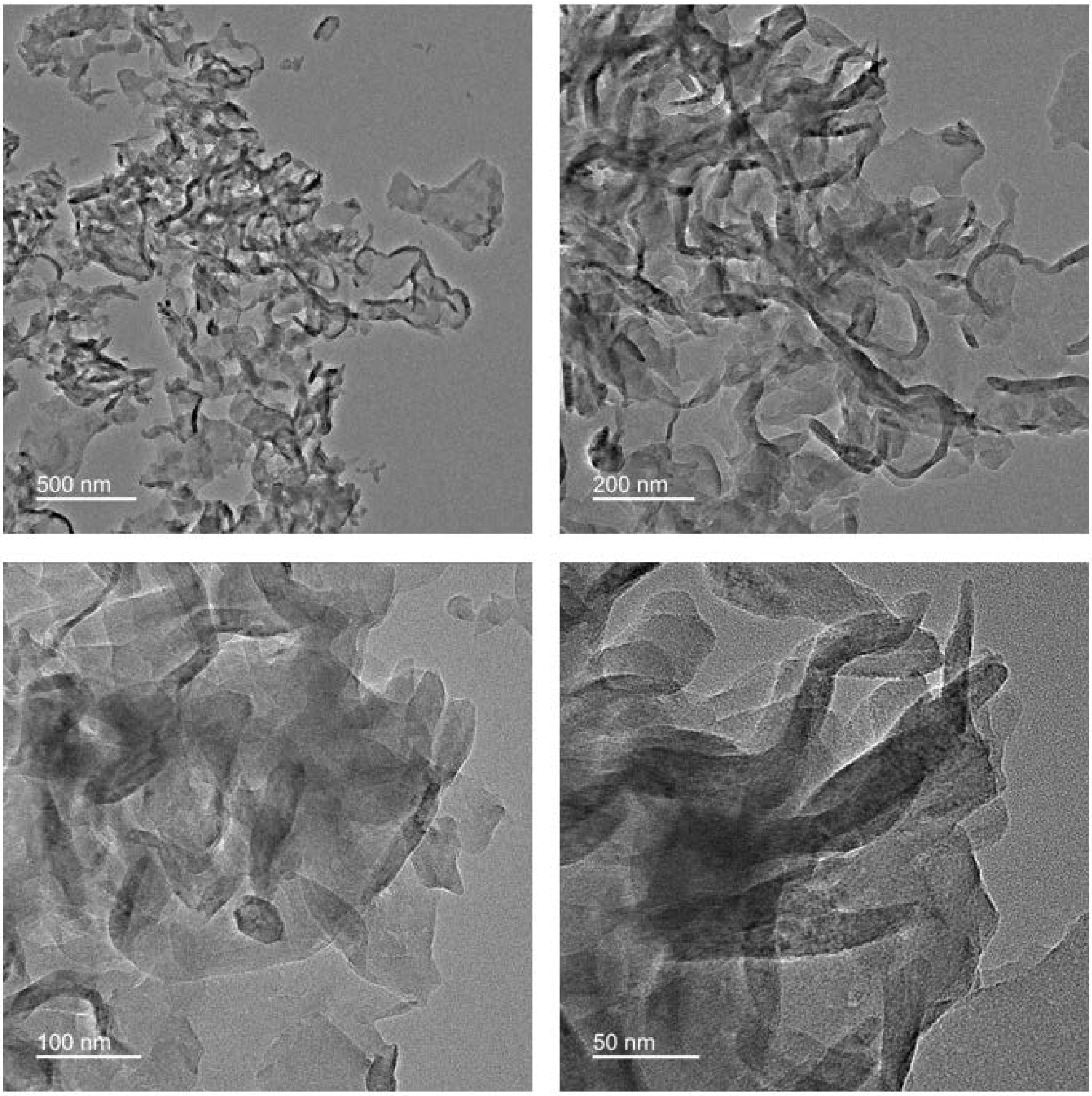

Fig. S5. TEM images of reference GCN.

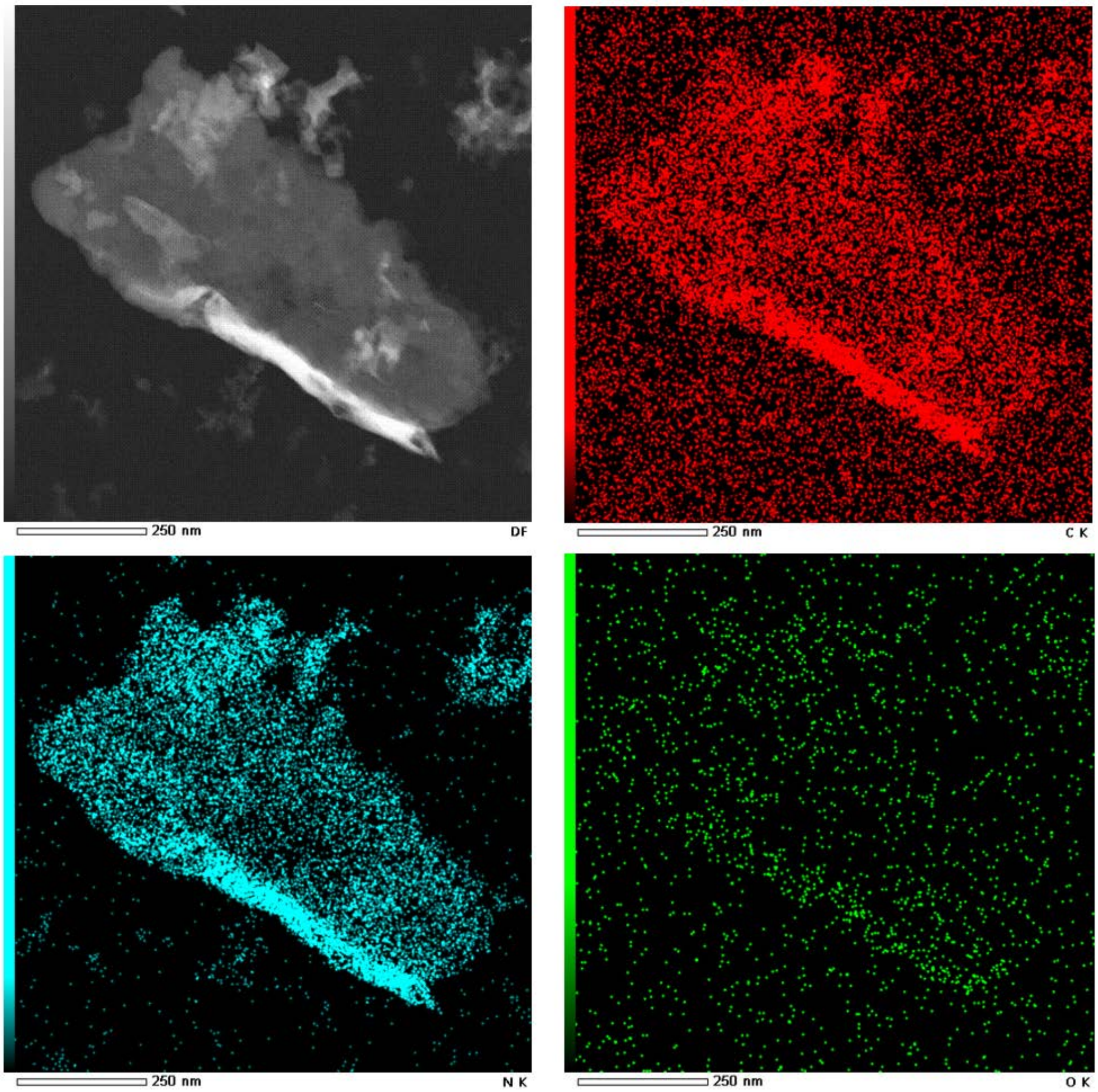

Fig. S6. EDX elemental maps of reference GCN.

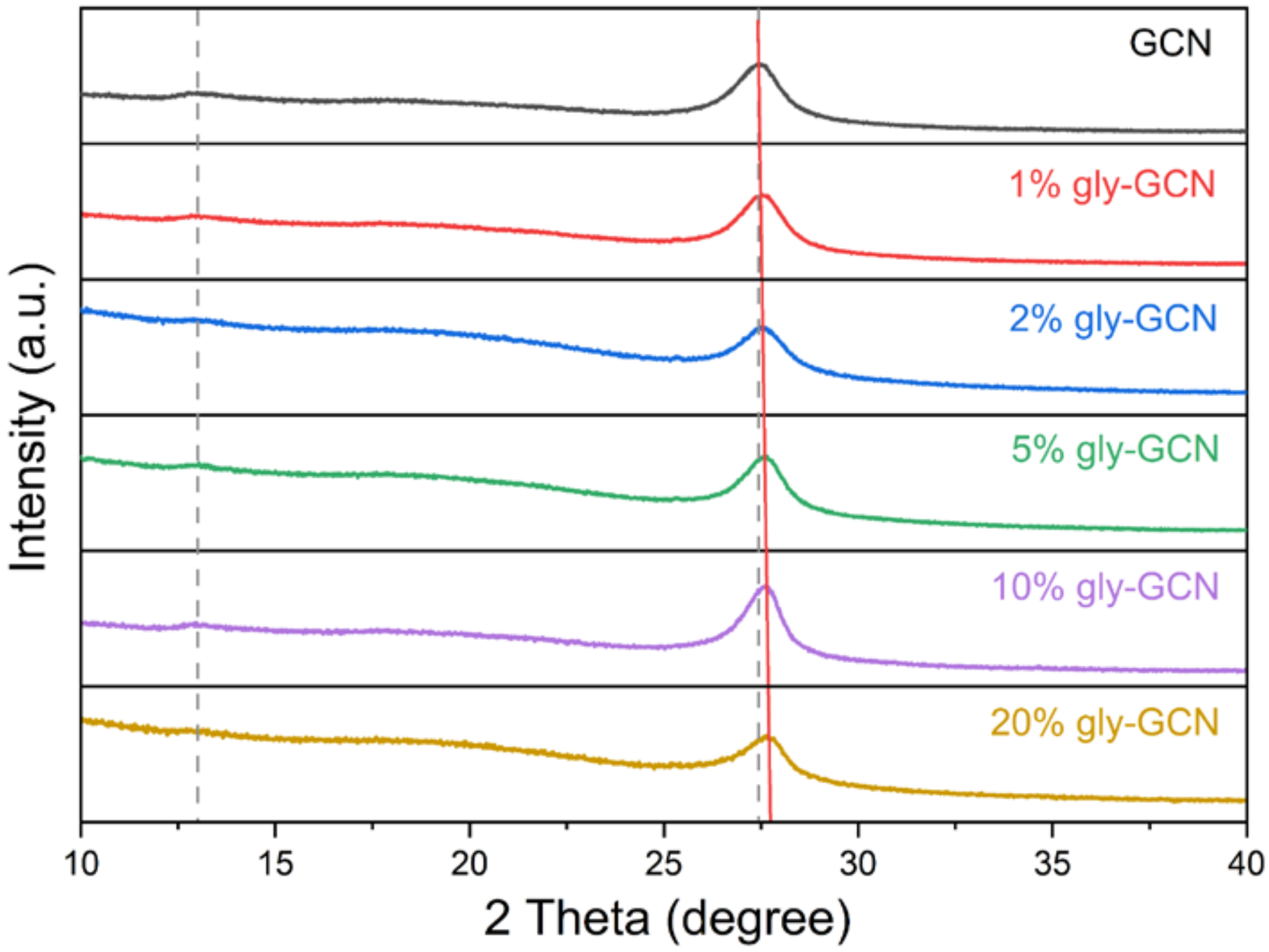

Fig. S7. XRD patterns of different GCN samples.

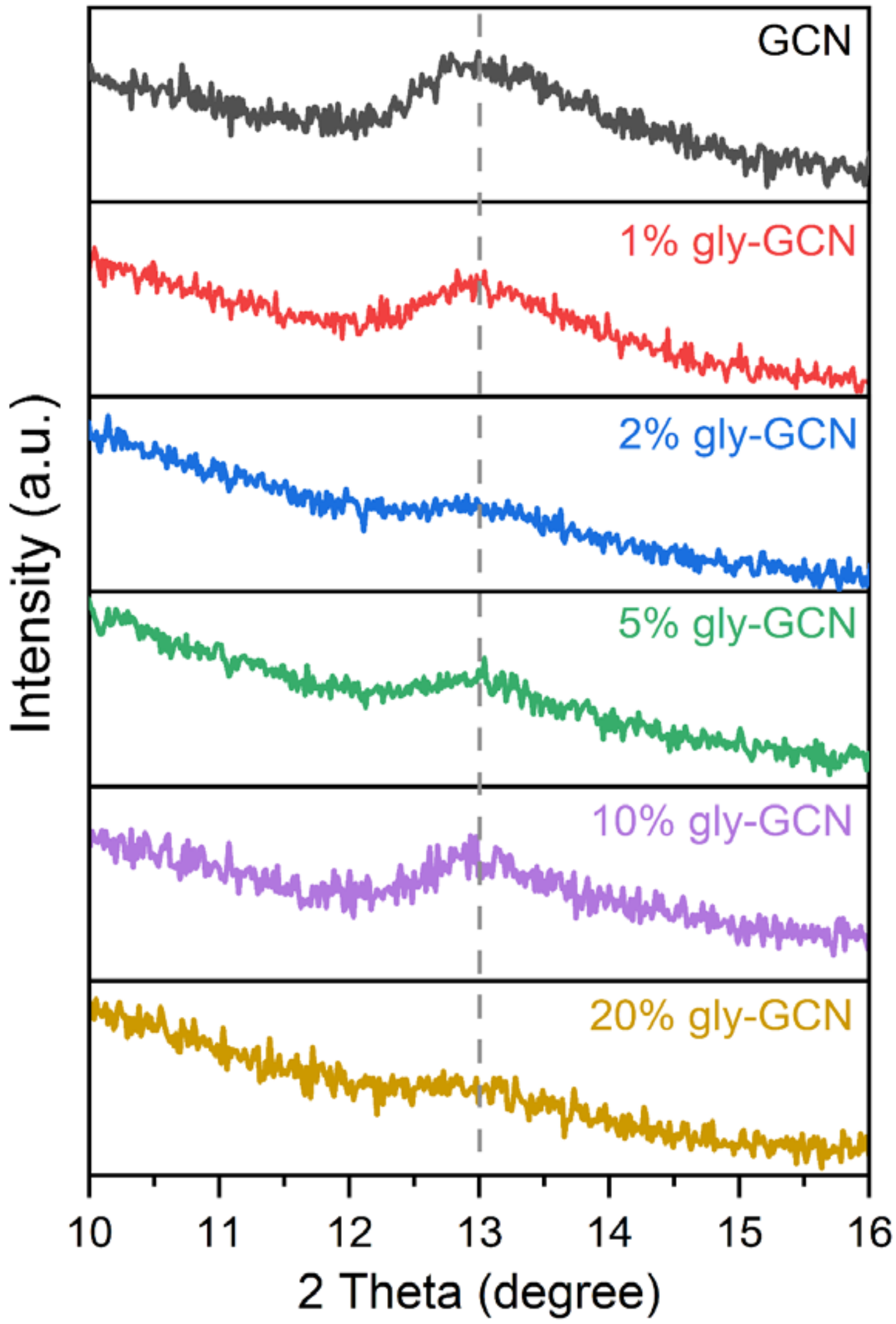

Fig. S8. XRD patterns of different samples in the 2θ range of 10-16°.

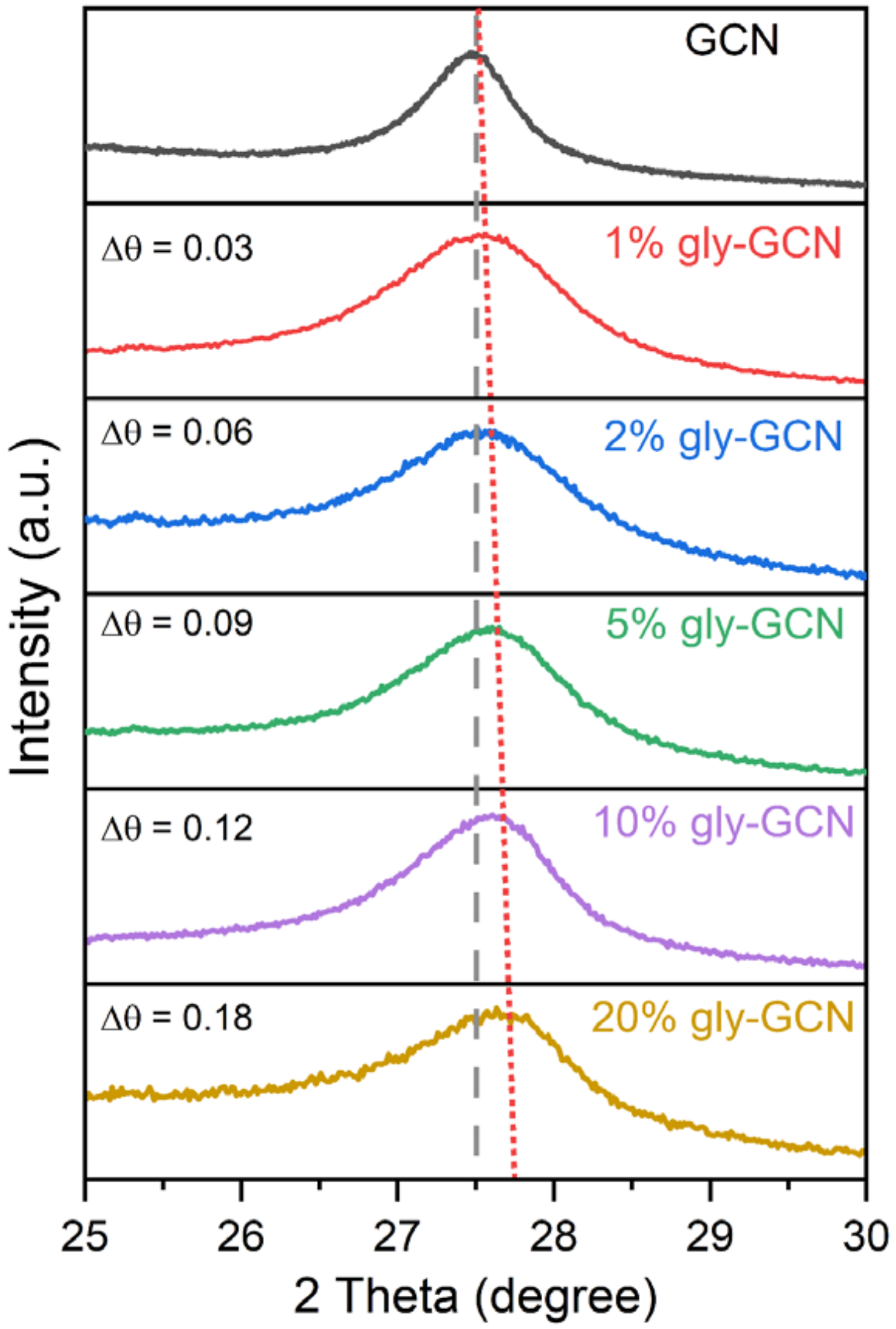

Fig. S9. XRD patterns of different samples in the 2θ range of 25-30°. The dashed vertical lines show the shift in the XRD peak positions.

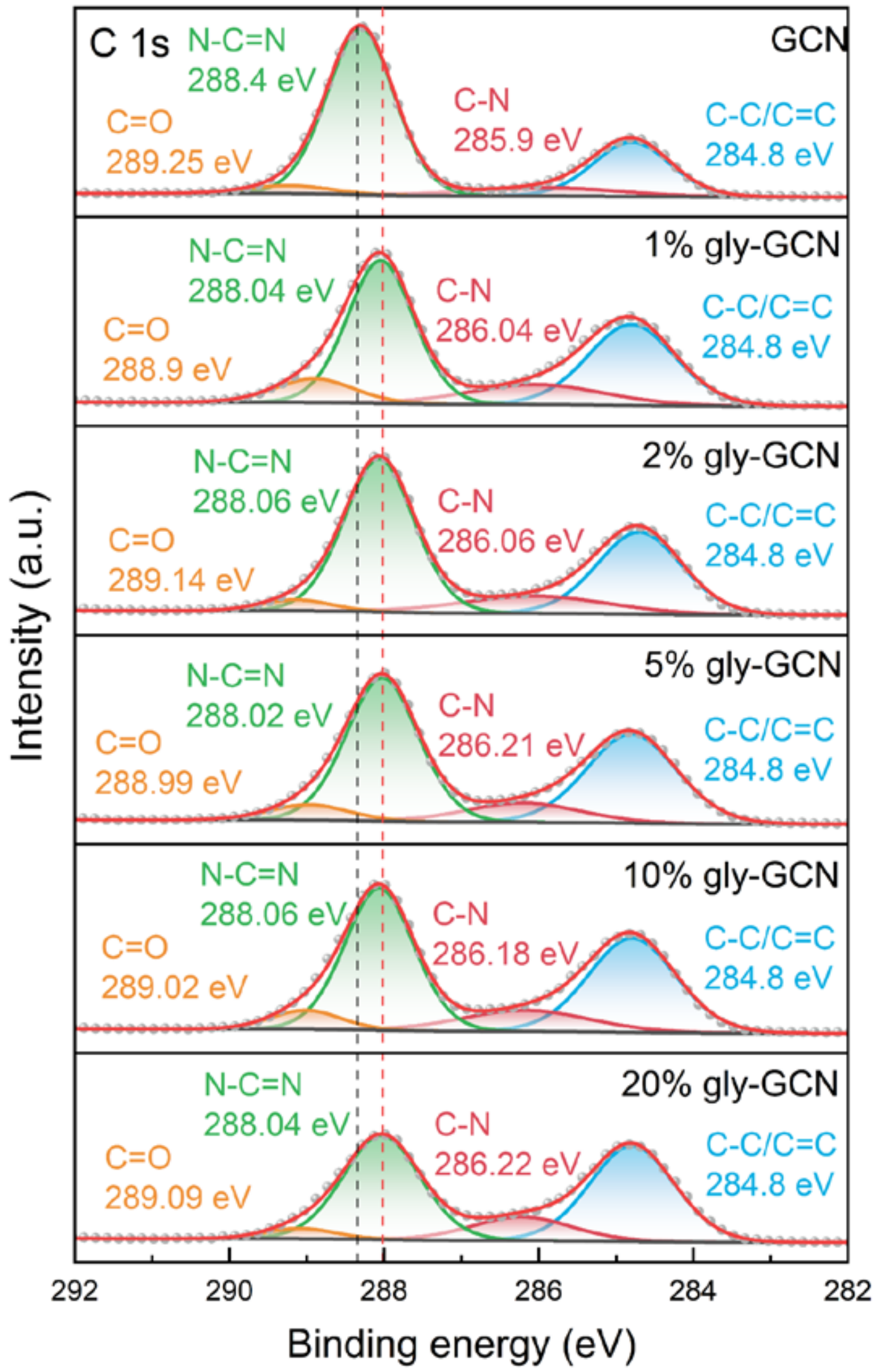

Fig. S10. High-resolution C 1s XP spectra of different GCN samples.

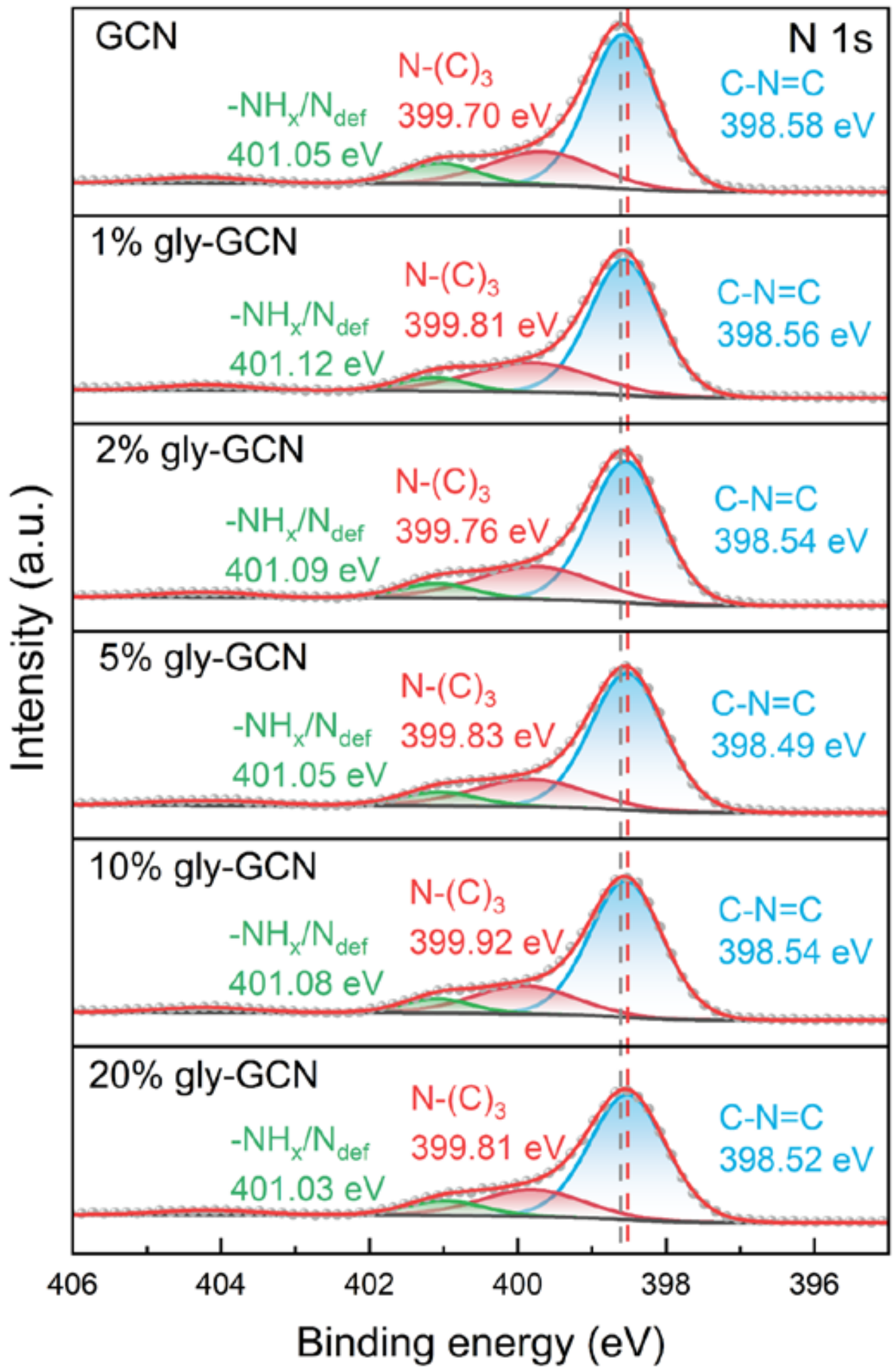

Fig. S11. High-resolution N 1s XP spectra of different GCN samples.

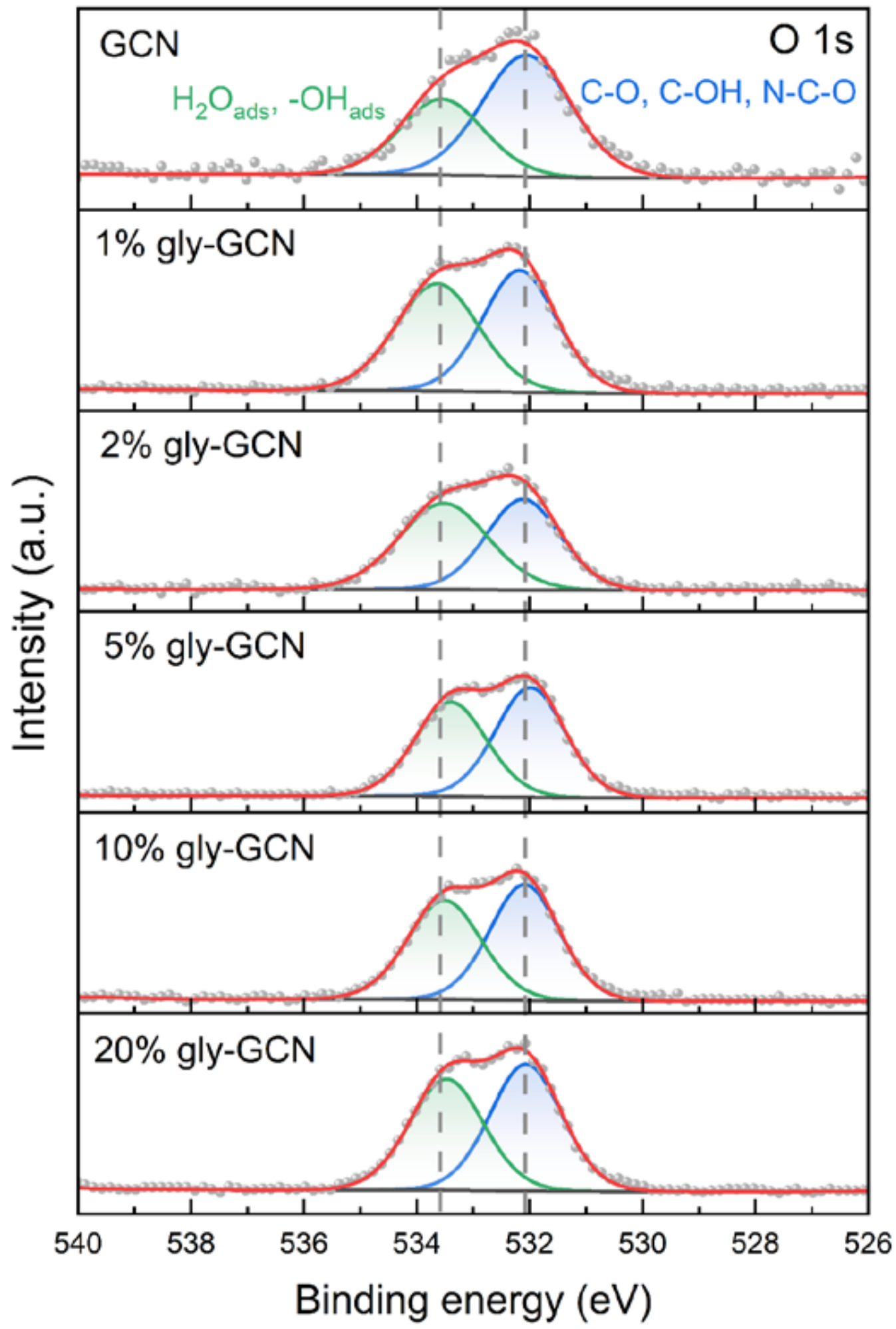

Fig. S12. High-resolution O 1s XP spectra of different GCN samples.

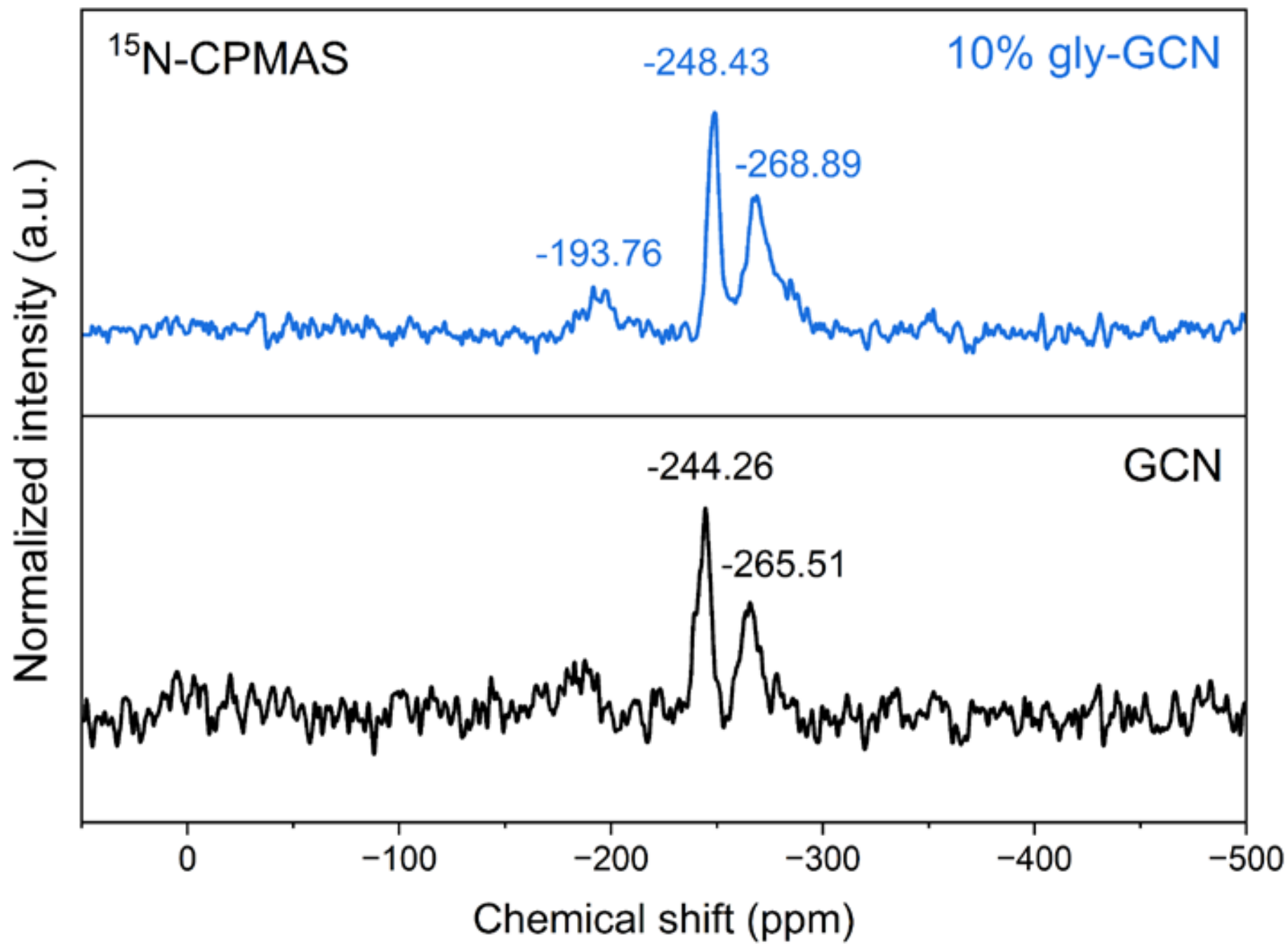

Fig. S13. ^{15}N CPMAS solid-state NMR spectra of GCN and 10% gly-GCN.

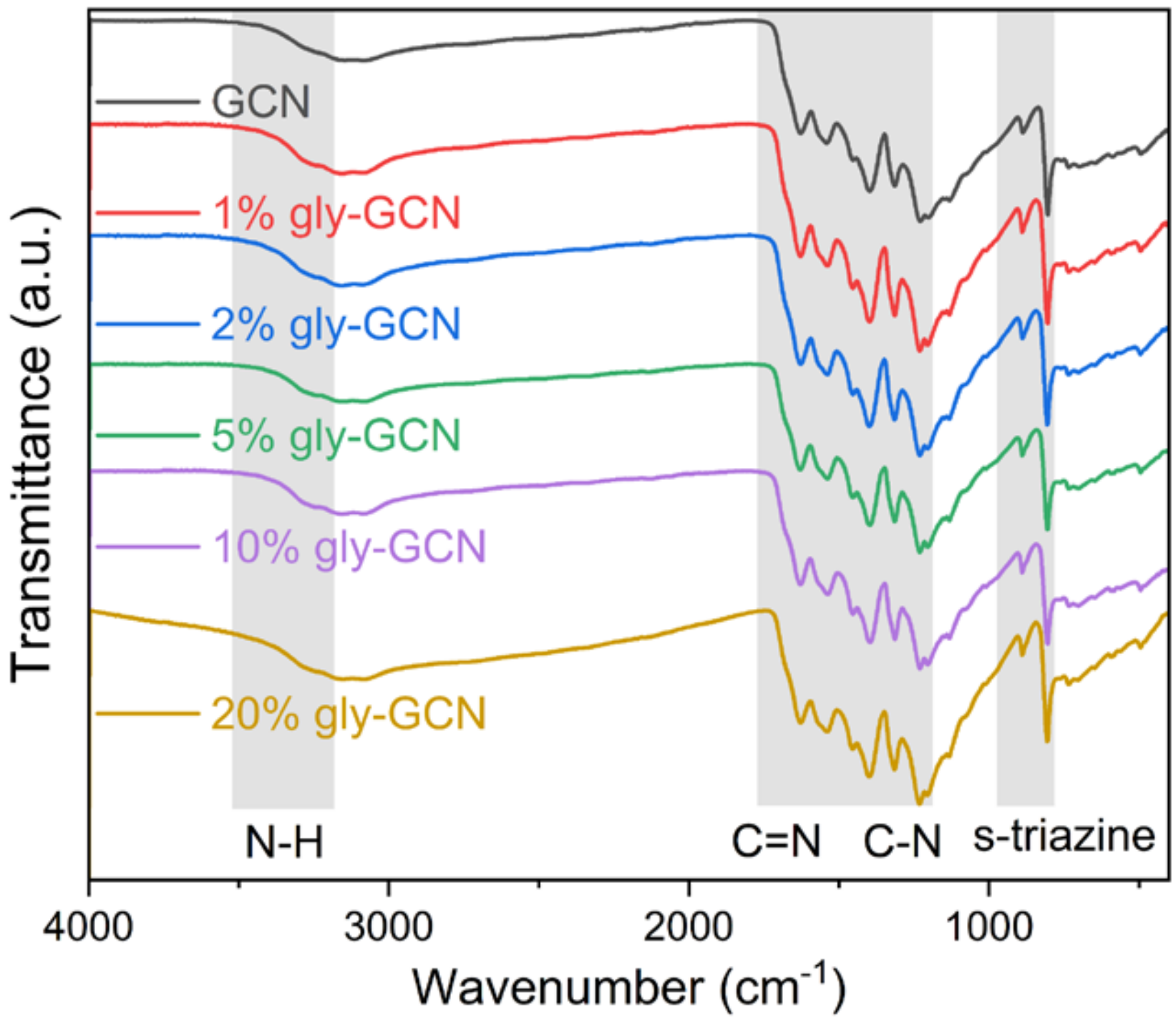

Fig. S14. FT-IR spectra of different GCN samples.

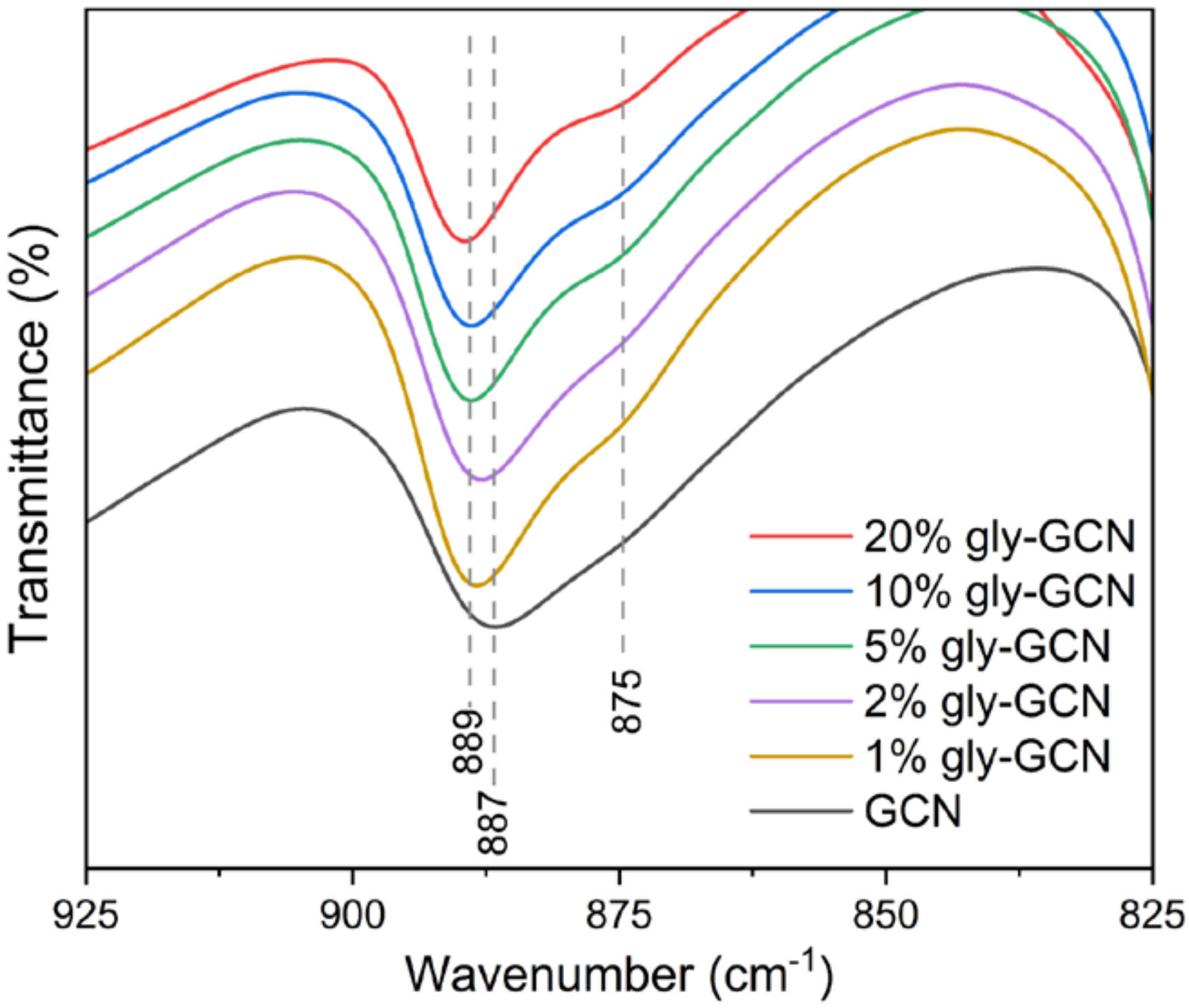

Fig. S15. Enlarged FT-IR spectra regions (zoom) of different GCN samples.

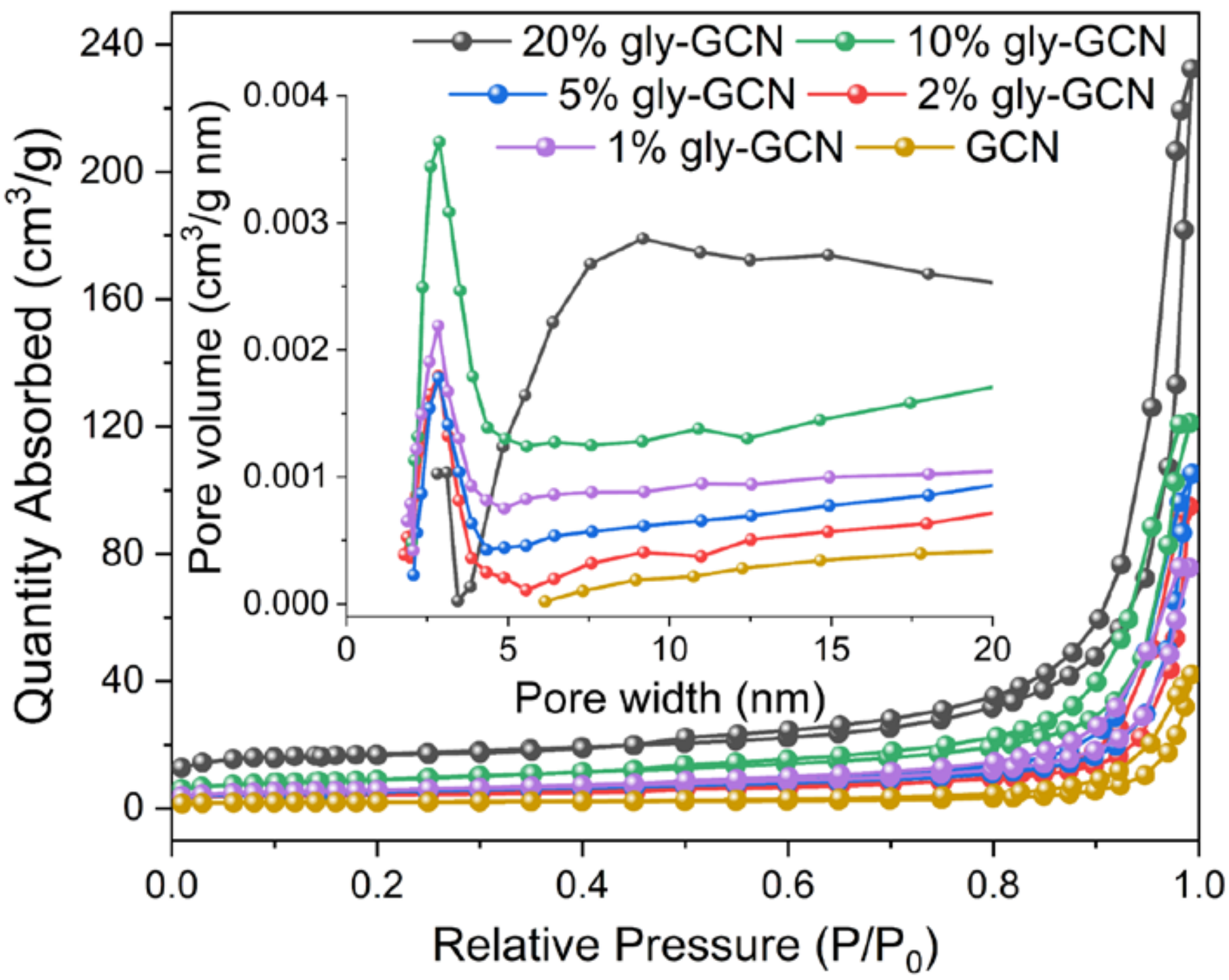

Fig. S16. N_2 adsorption-desorption isotherms and the corresponding pore size distribution (inset) of the different GCN samples.

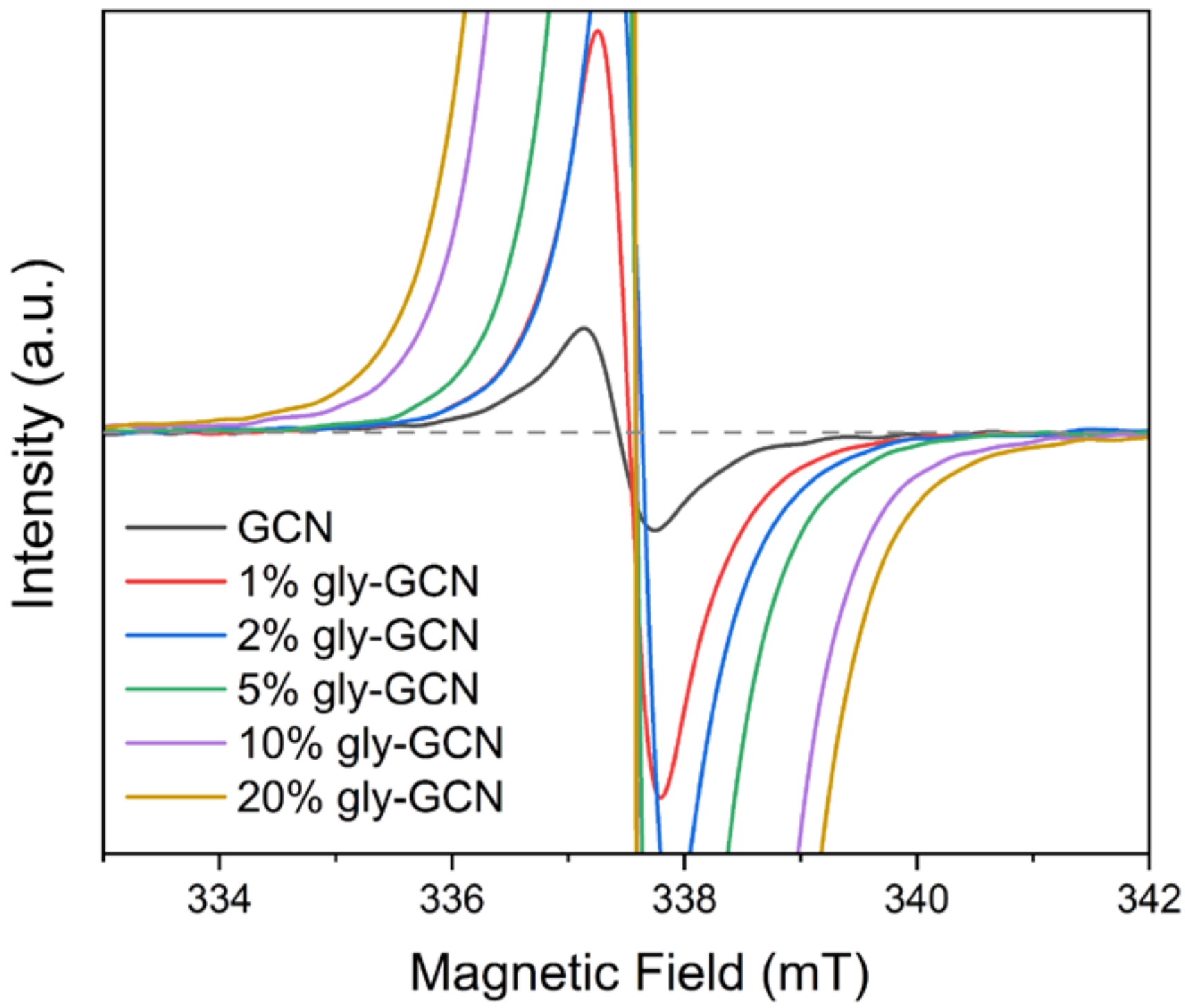

Fig. S17. Enlarged EPR spectra (zoom) of different GCN samples.

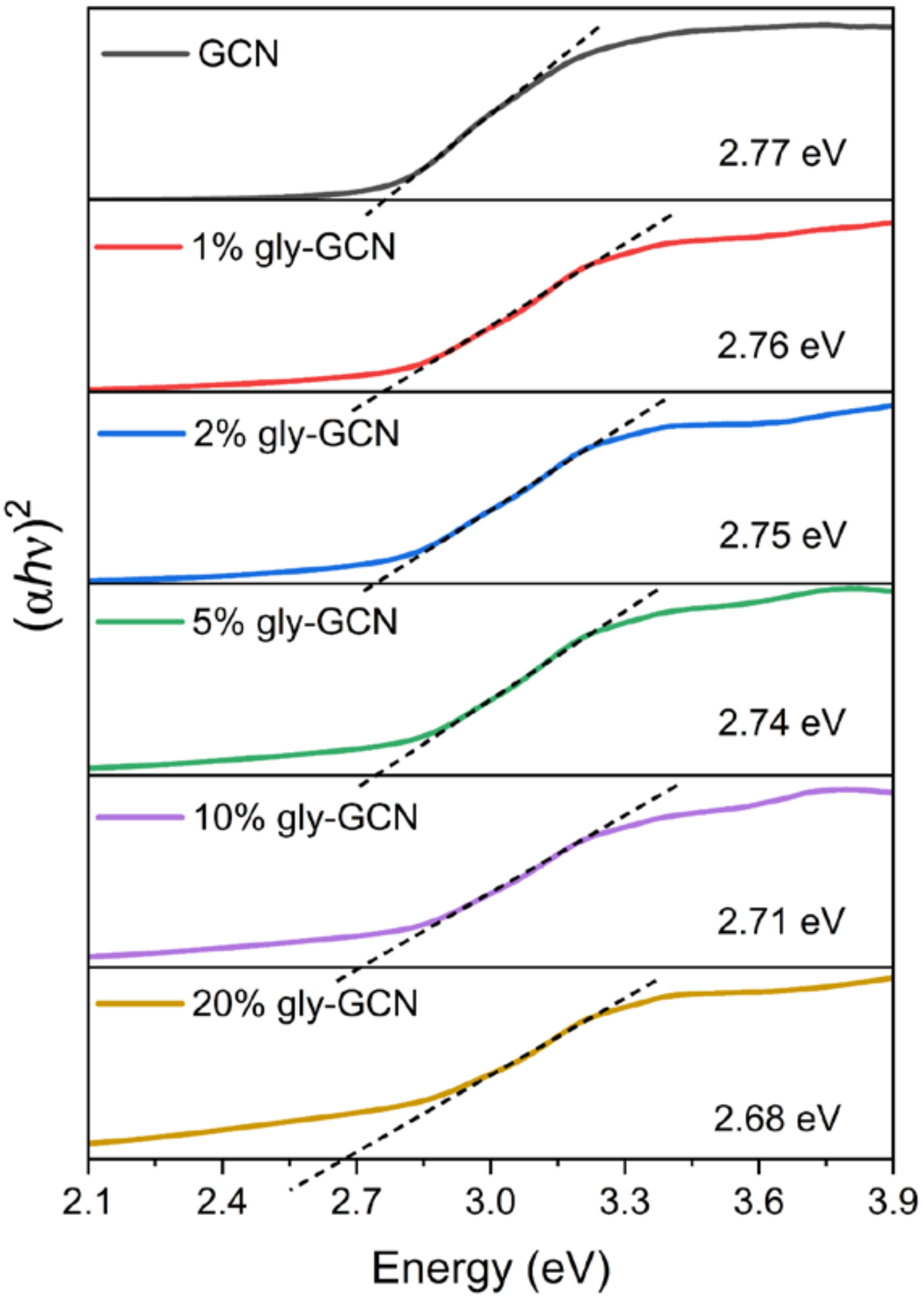

Fig. S18. Tauc plots of different GCN samples.

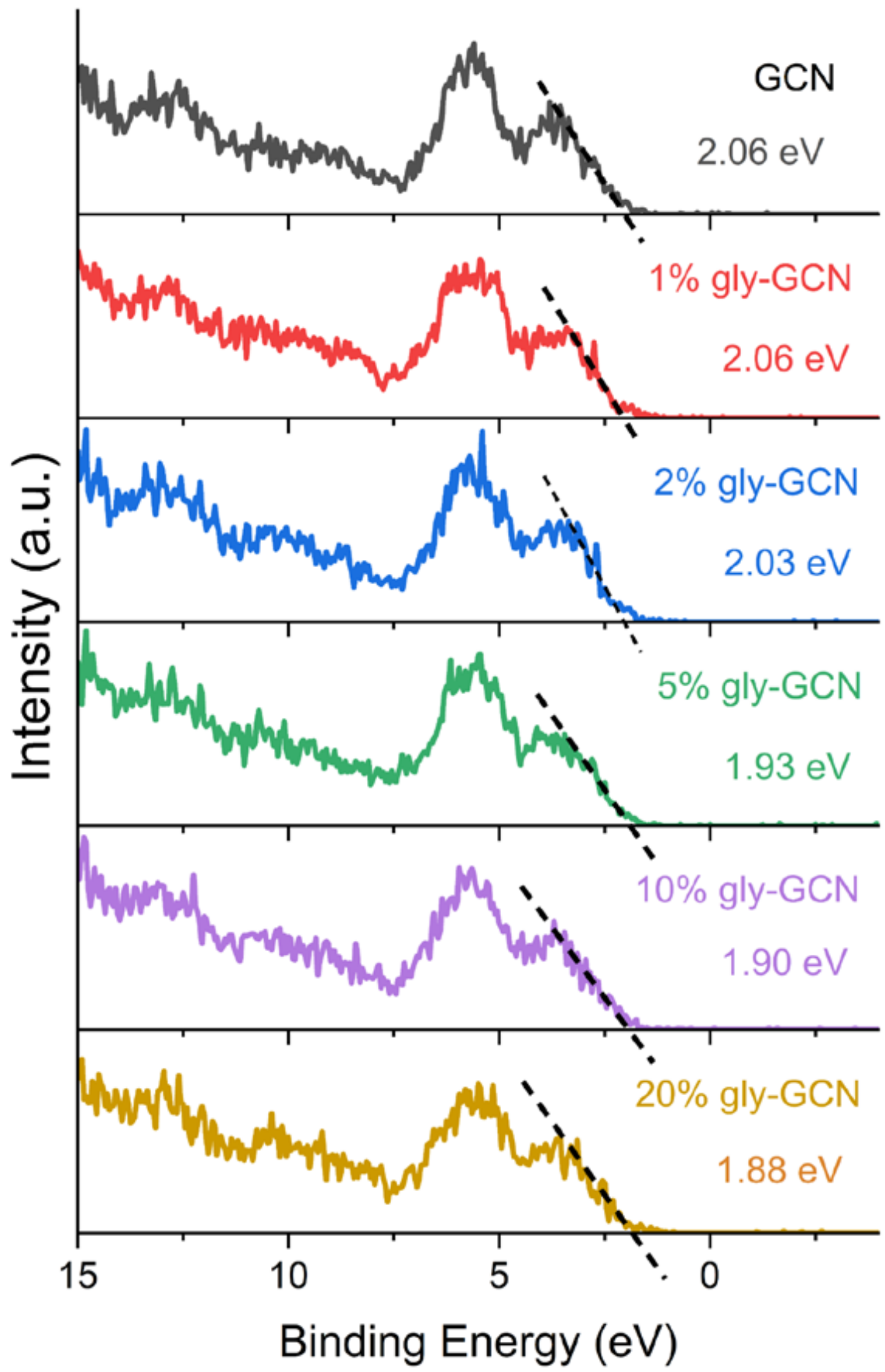

Fig. S19. XP valence band spectra of the different GCN samples.

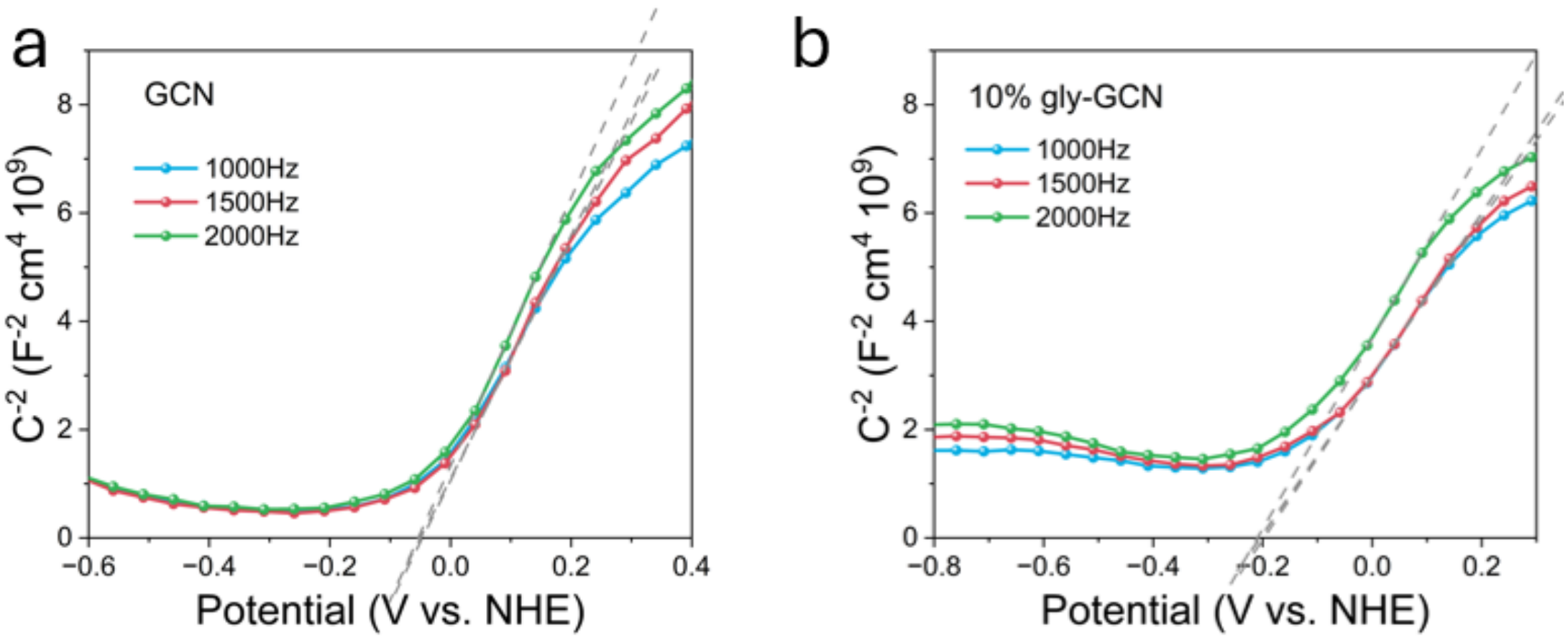

Fig. S20. Mott-Schottky plots measured at different frequencies for (a) GCN and (b) 10% gly-GCN.

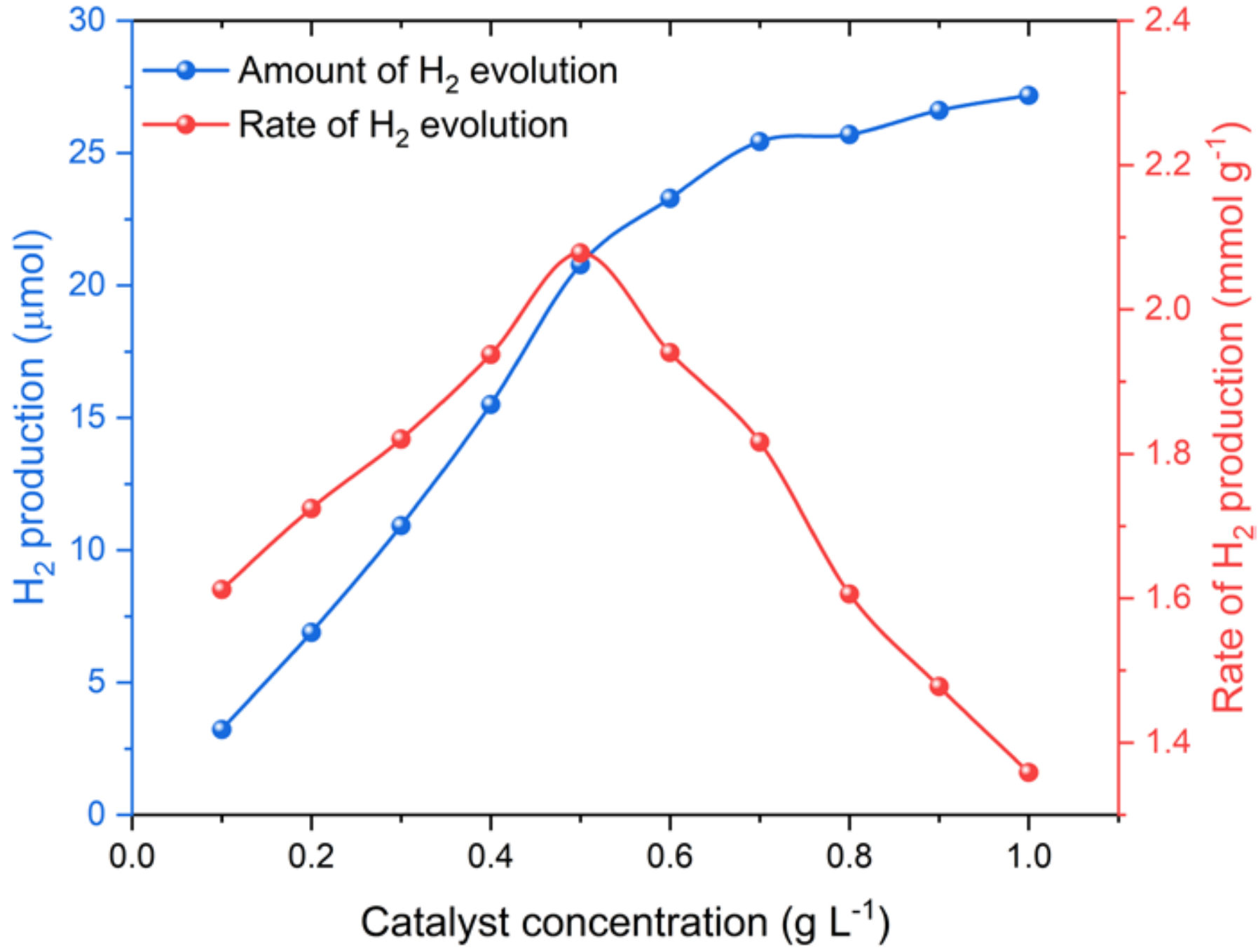

Fig. S21. Optimization of 10% gly-GCN concentration for photocatalytic H_2 evolution. The blue line is the H_2 amount at different catalyst concentrations, and the red line is the corresponding H_2 evolution rate.

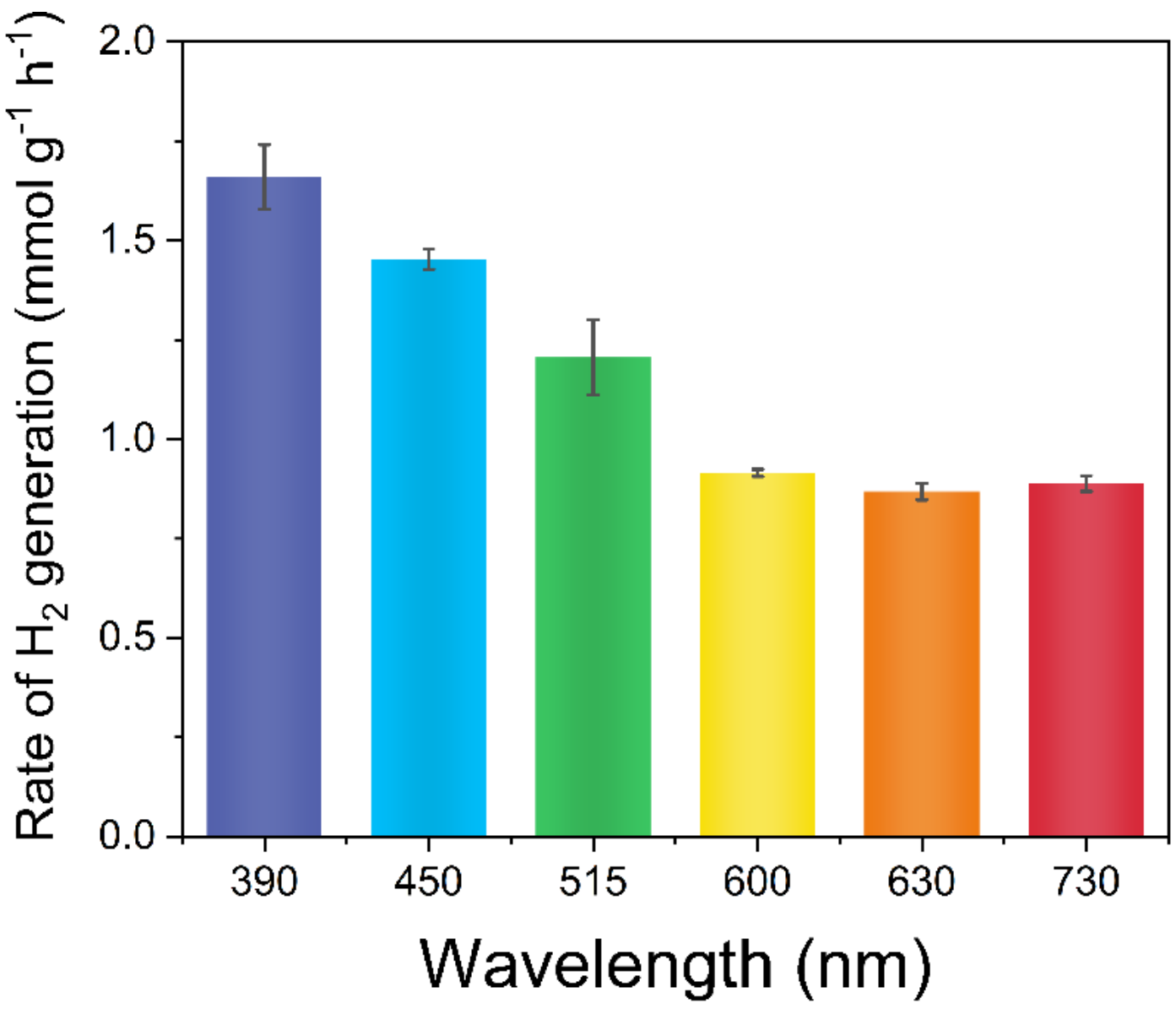

Fig. S22. Photocatalytic H_2 evolution efficiency of the 10% gly-GCN sample at different wavelengths, used for calculating the AQY.

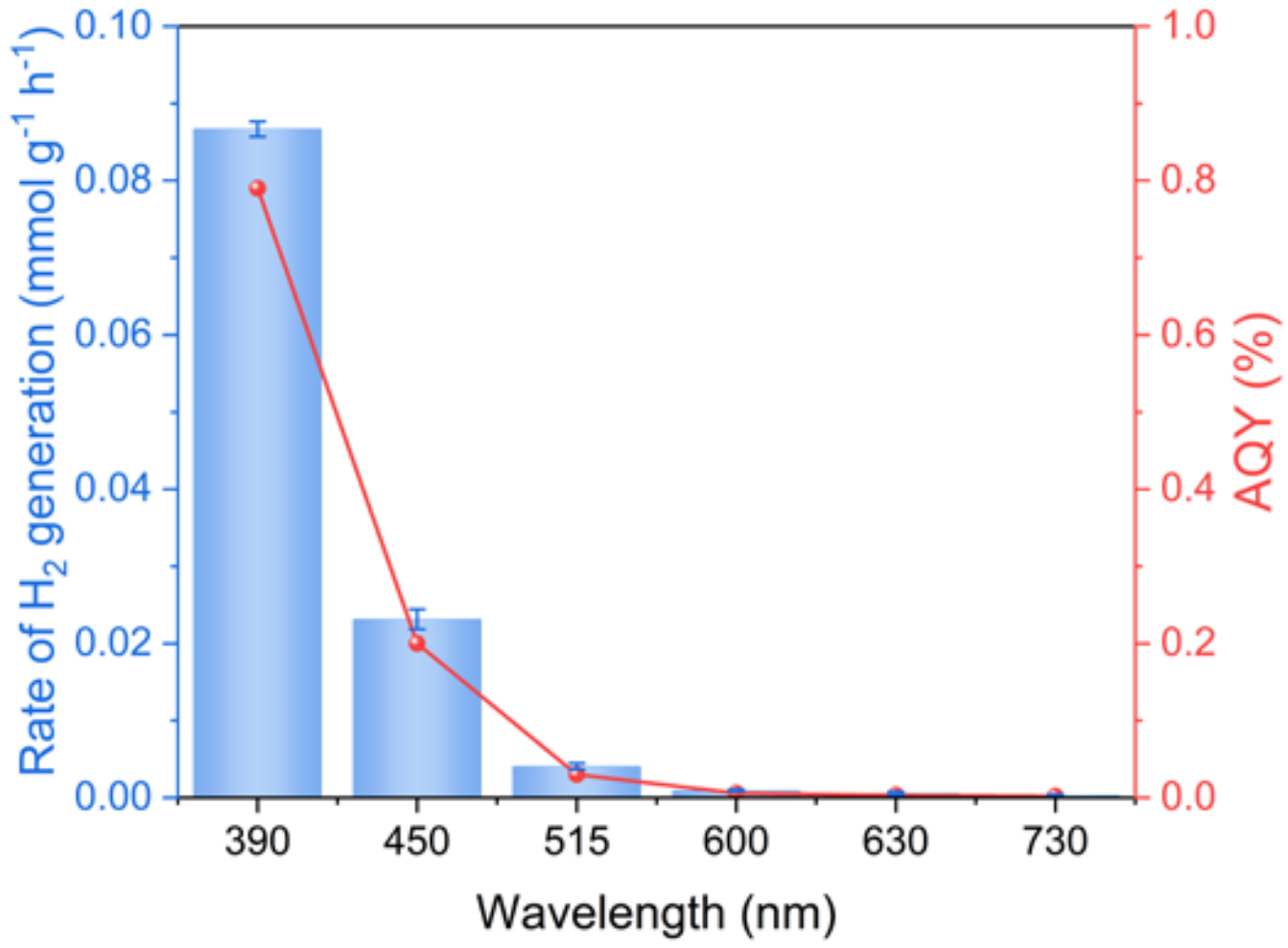

Fig. S23. Photocatalytic H_2 evolution efficiency of reference GCN sample at different wavelengths (blue) and calculated AQY at corresponding wavelengths (red).

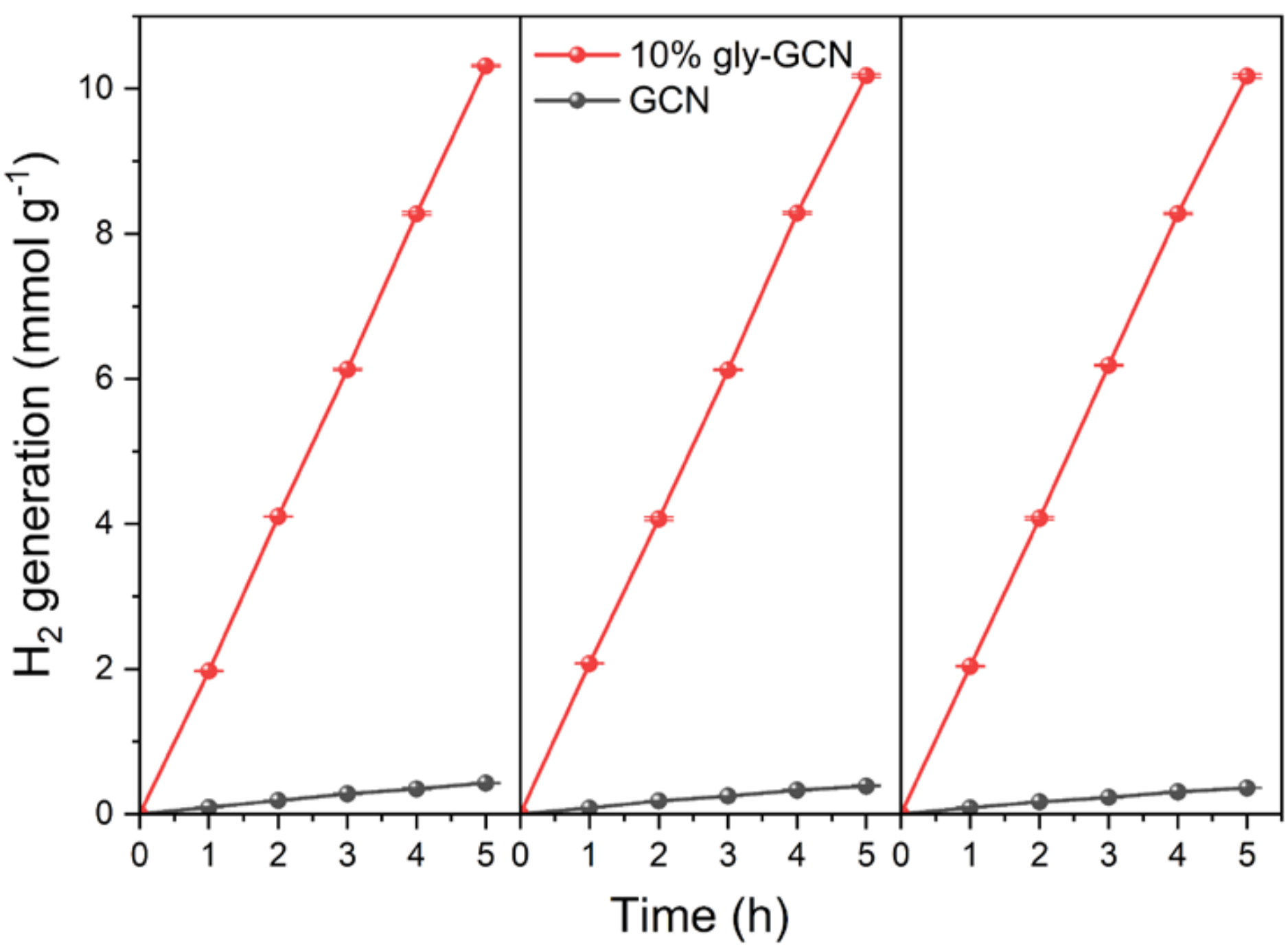

Fig. S24. Cycling stability of 10% gly-GCN and GCN for photocatalytic H_2 evolution.

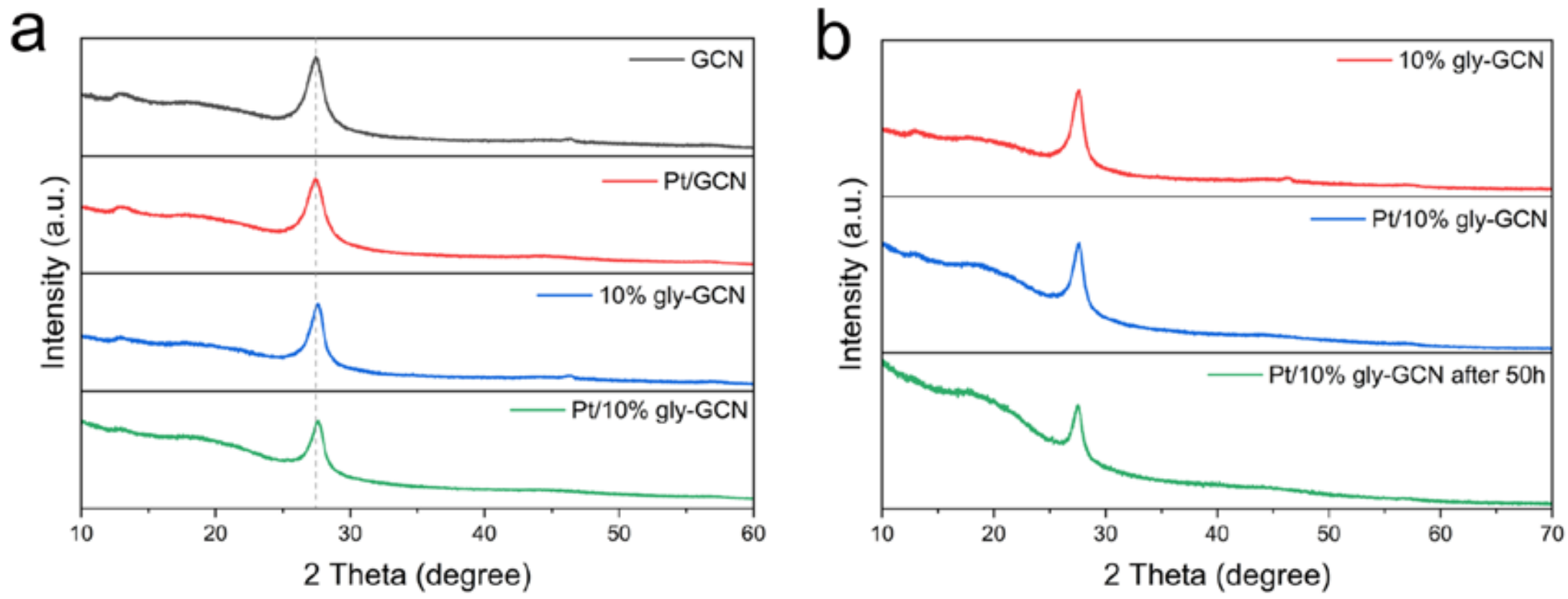

Fig. S25. (a) PXRD patterns of GCN and 10% gly-GCN before and after Pt deposition. (b) PXRD patterns of 10% gly-GCN, Pt/10% gly-GCN and Pt/10% gly-GCN after 50 h photocatalytic H_2 evolution.

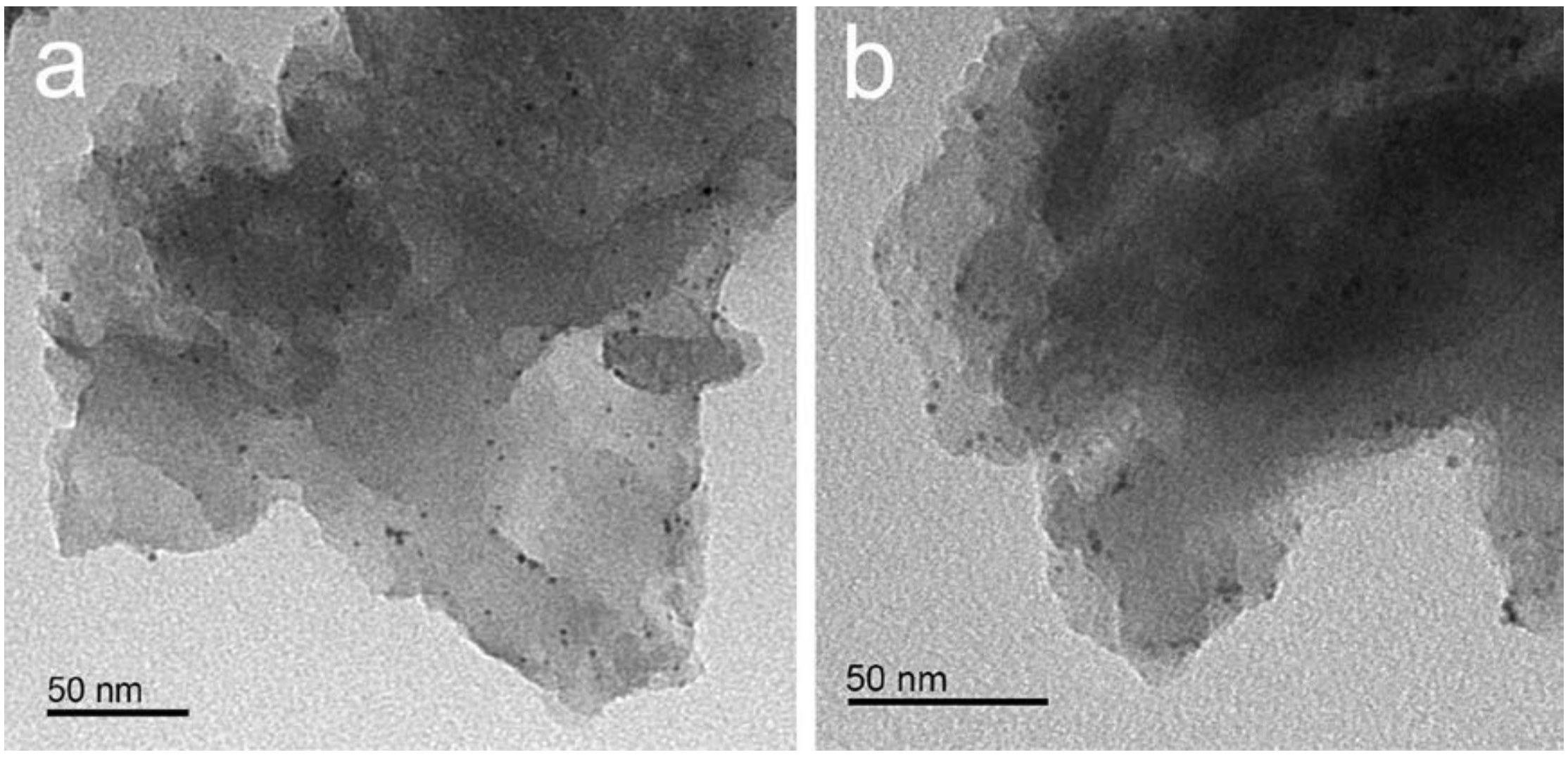

Fig. S26. TEM images of (a) Pt/10% gly-GCN and (b) Pt/10% gly-GCN after 50 h photocatalytic H_2 evolution.

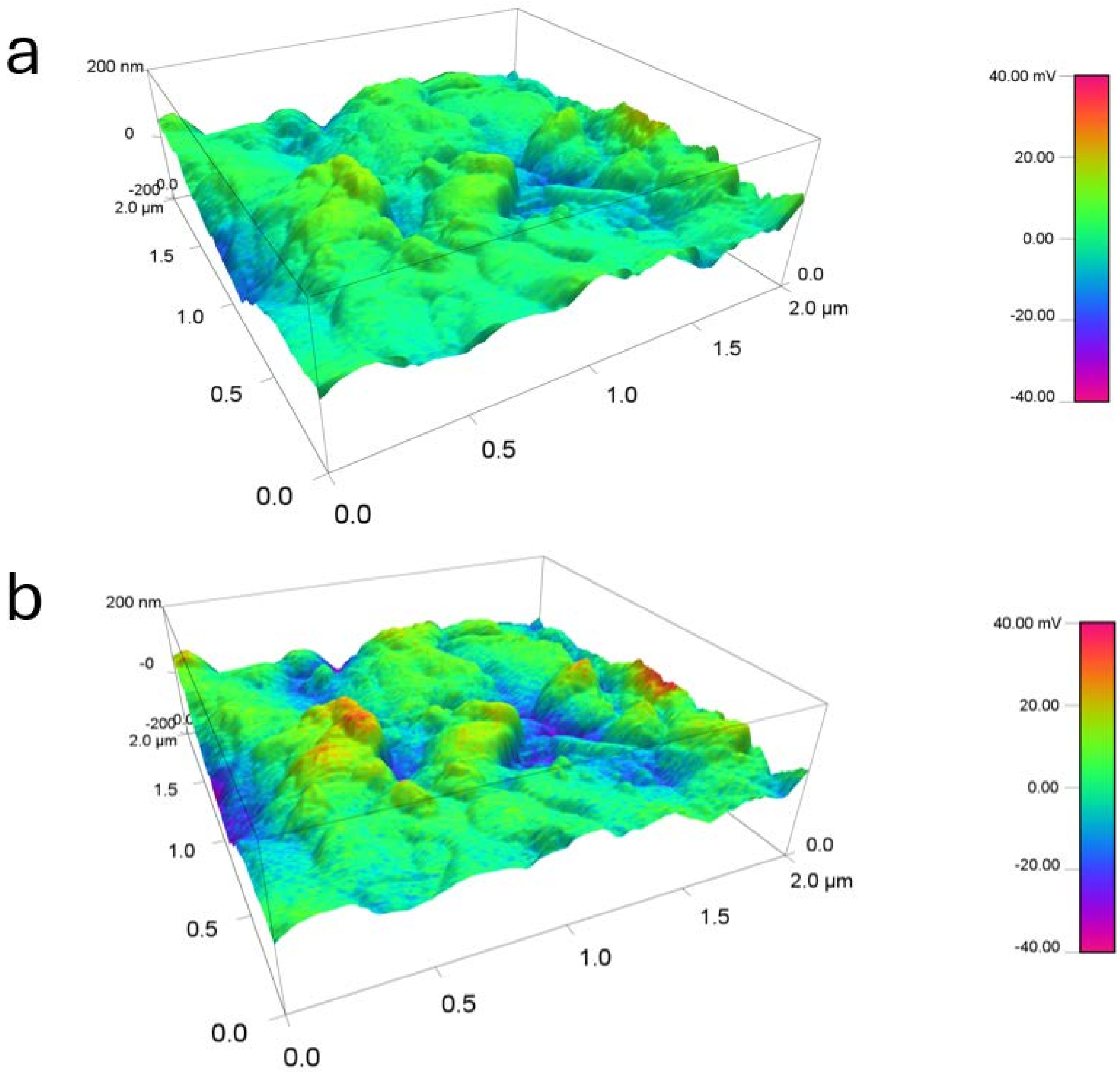

Fig. S27. Three-dimensional topography maps of reference GCN sample obtained from AFM-KPFM measurements, where the height axis represents AFM surface height variations and the color mapping indicates the CPD values measured by KPFM: (a) under dark conditions and (b) under illumination.

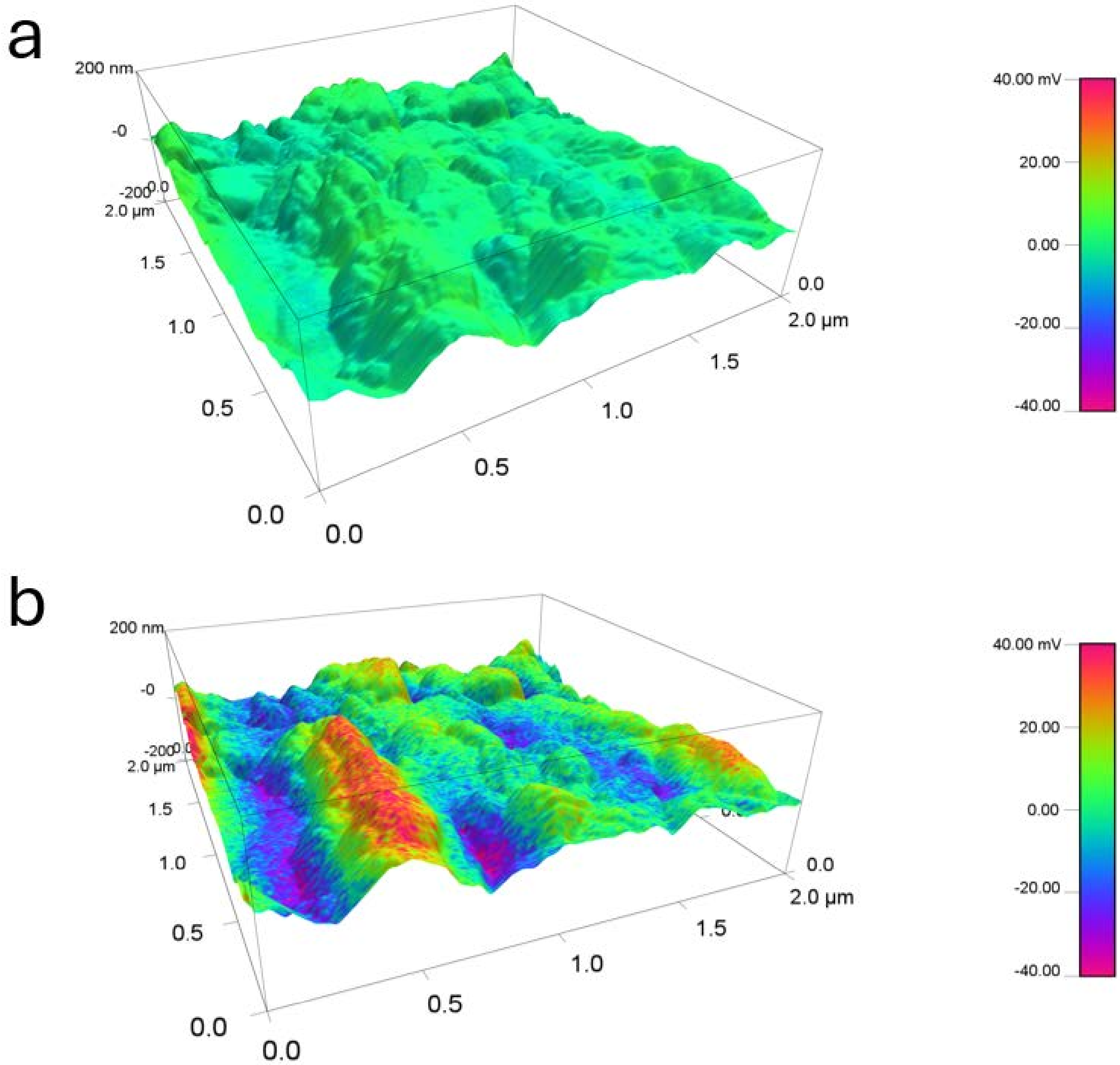

Fig. S28. Three-dimensional topography maps of the 10% gly-GCN sample obtained from AFM-KPFM measurements, where the height axis represents AFM surface height variations and the color mapping indicates the CPD values measured by KPFM: (a) under dark conditions and (b) under light illumination.

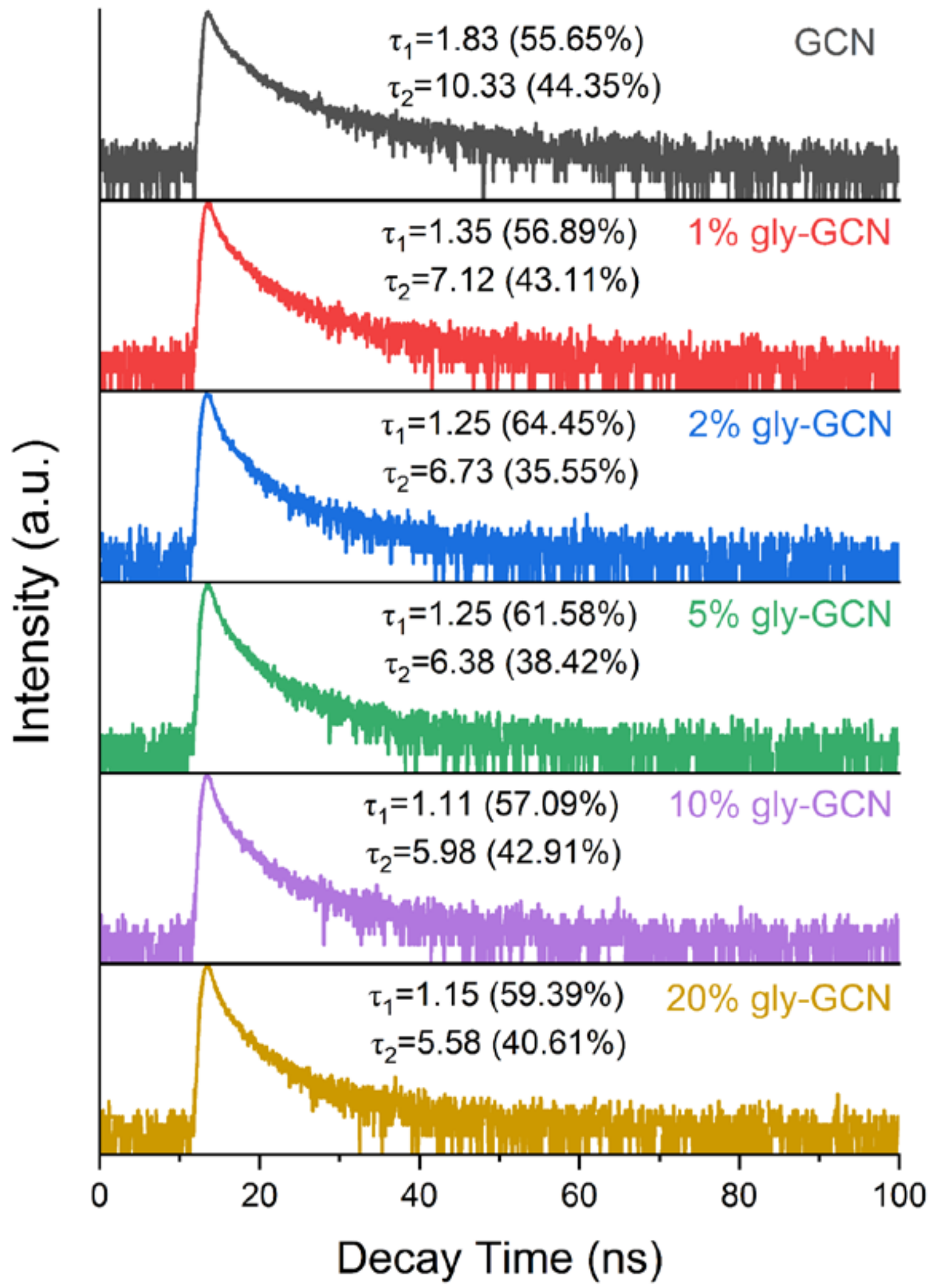

Fig. S29. TRPL spectra of different GCN powder samples.

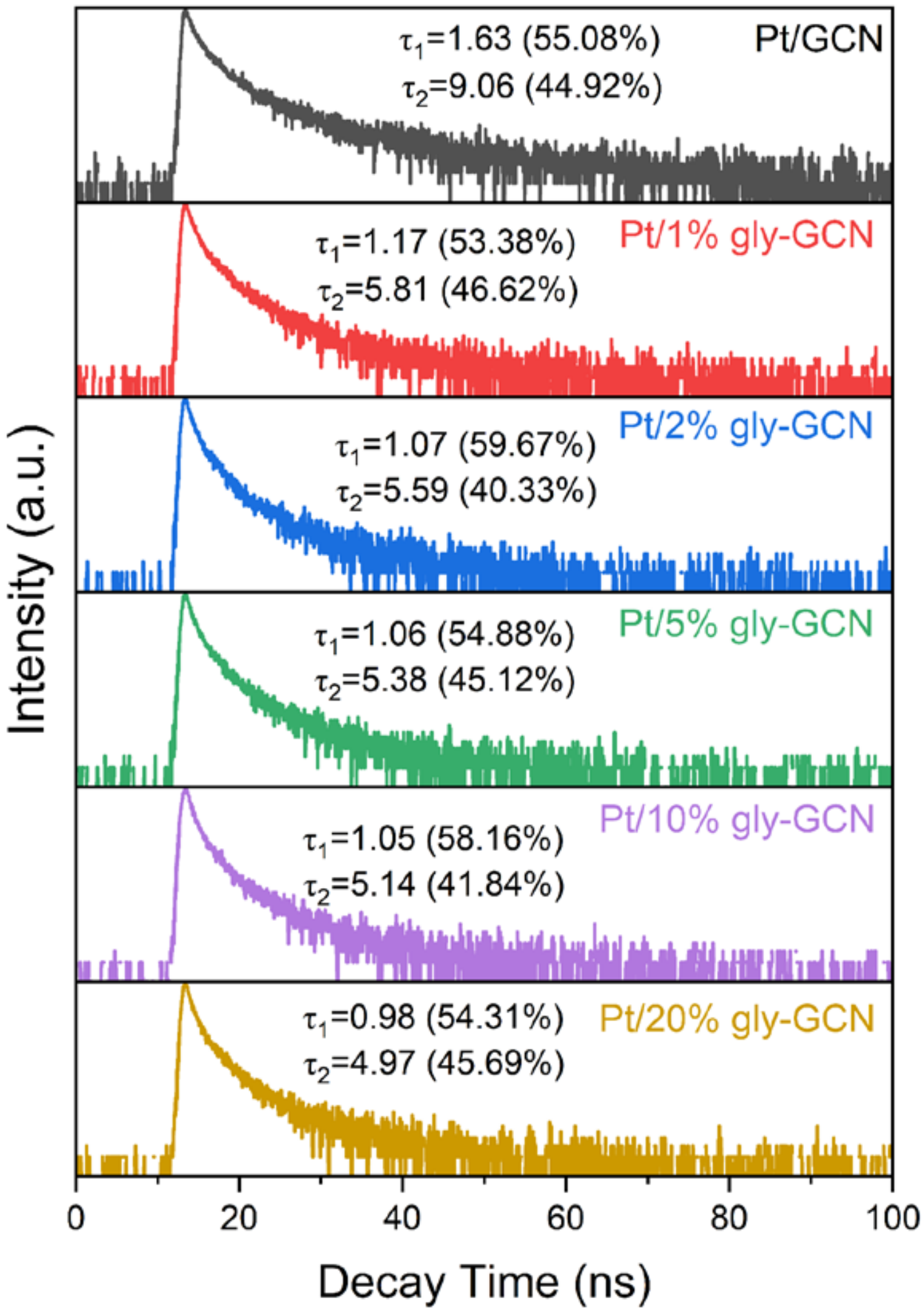

Fig. S30. TRPL spectra of different samples measured under photocatalytic hydrogen evolution conditions.

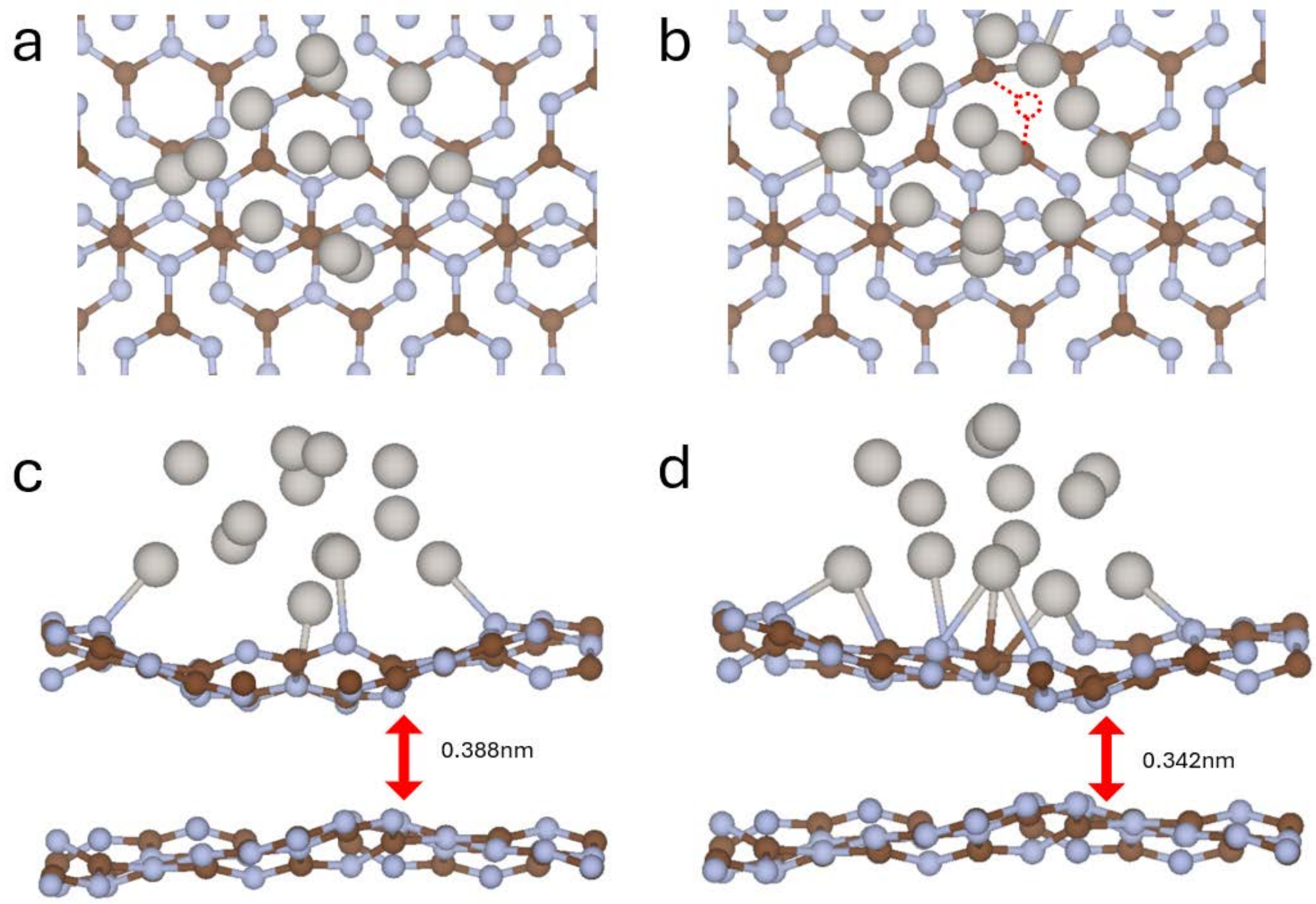

Fig. S31. Calculated models of Pt/GCN and Pt/N_v-GCN: (a) top view of Pt/GCN and (b) top view of Pt/GCN-nar, where red dashed lines indicate N vacancies; (c) interlayer spacing of Pt/GCN and (d) interlayer spacing of Pt/N_v-GCN.

Table S1. C/H/N/S/O elemental analysis results of different GCN and gly-GCN samples.

**Samples**	**C (wt.%)**	**H (wt.%)**	**N (wt.%)**	**O (wt.%)**	**S (wt.%)**
**GCN**	34.22±0.04	1.94±0.02	60.53±0.07	3.31±0.18	<0.2
**1% gly-GCN**	34.35±0.04	1.85±0.01	60.44±0.06	3.43±0.16	<0.2
**2% gly-GCN**	34.47±0.05	1.79±0.01	60.23±0.11	3.53±0.08	<0.2
**5% gly-GCN**	34.87±0.06	1.92±0.01	59.65±0.09	3.57±0.06	<0.2
**10% gly-GCN**	35.42±0.01	1.816±0.02	58.17±0.05	4.74±0.15	<0.2
**20% gly-GCN**	36.94±0.07	2.1±0.01	54.63±0.11	6.33±0.11	<0.2

Table S2. Calculations of C/N, H/N, O/N, and C/H/N/O ratios for different gly-GCN samples based on C/H/N/S/O elemental analysis results.

Samples	C/N (at. %)	H/N (at. %)	O/N (at. %)	C/H/N/O (at. %)
**GCN**	0.661	0.437	0.050	$C_3H_{1.98}N_{4.54}O_{0.23}$
**1% gly-GCN**	0.663	0.423	0.050	$C_3H_{1.92}N_{4.52}O_{0.22}$
**2% gly-GCN**	0.669	0.411	0.052	$C_3H_{1.84}N_{4.48}O_{0.23}$
**5% gly-GCN**	0.684	0.450	0.053	$C_3H_{1.98}N_{4.39}O_{0.23}$
**10% gly-GCN**	0.707	0.420	0.071	$C_3H_{1.78}N_{4.24}O_{0.30}$
**20% gly-GCN**	0.791	0.533	0.103	$C_3H_{2.02}N_{3.79}O_{0.39}$

Table S3. BET analysis of different GCN and gly-GCN samples.

**Materials**	**BET Surface Area (m^2/g)**	**Micropore Area (m^2/g)**	**Mesopore Area (m^2/g)**	**Total Pore Volume (cm^3/g)**
**GCN**	7.4	<0.002	8.403	0.065
**1% gly-GCN**	12.6	<0.002	19.254	0.059
**2% gly-GCN**	14.6	<0.002	22.828	0.068
**5% gly-GCN**	13.1	<0.002	21.623	0.059
**10% gly-GCN**	20.1	<0.002	22.418	0.118
**20% gly-GCN**	41.6	<0.002	61.139	0.125

Table S4. Comparison of the photocatalytic H_2 evolution performance of the investigated GCN-based catalysts with recent representative studies. The factor 'Improved H_2 production' is defined as the ratio of the H_2 evolution rate of each modified catalyst to that of its corresponding unmodified GCN reference measured under the same experimental conditions within the original study. Therefore, this factor reflects the relative improvement achieved in each individual study and is independent of the absolute H_2 evolution rate.

Sample	Co-catalyst	Sacrificial agent	Light source	Photocatalysis H_2 performance (mmol h^{-1} g^{-1})	Improved H_2 production (times)	Ref.
**10% gly-GCN**	3.0 wt.% Pt	TEOA	LED visible light illumination	2.07	23	This work
**g-C_3N_4 nanomesh**	3.0 wt.% Pt	TEOA	300 W Xe lamp	8.51	24.3	[10]
**GD-C_3N_4**	3.0 wt.% Pt	TEOA	300 W Xe lamp	2.31	21	[11]
**O, P-CNS**	3.0 wt.% Pt	TEOA	LED visible light illumination	8.76	10	[12]
**DACN-100**	3.0 wt.% Pt	TEOA	300 W Xe lamp	37.48	8.6	[13]
**NO-CNNS**	1 wt.% Pt	TEOA	300 W Xe lamp	5.7	5	[14]
**O-CN/CN-3**	3.0 wt.% Pt	TEOA	300 W Xe lamp	6.97	12.4	[15]
**CNPS-NH_2**	3.0 wt.% Pt	TEOA	300 W Xe lamp	20.95	3.9	[16]
**1.8PCN**	None	Lactic acid	300 W Xe lamp	0.57	13.3	[17]
**H-PHI**	2.0 wt.% Pt	Methanol	300 W Xe lamp	3.36	38	[18]
**BPCN**	1 wt. % Pt	TEOA	300 W Xe lamp	4.58	31.6	[19]
**BQCN**	3.0 wt.% Pt	TEOA	300 W Xe lamp	1.99	1.3	[20]
**Mel-PHI**	4.0 wt.% Pt	TEOA	300 W Xe lamp	5.6	12.4	[21]

**bC_3N_4**	3.75 wt.% Pt	TEOA	300 W Xe lamp	21.35	1.7	[22]
**NCD-CN**	2 wt.% Pt	TEOA	300 W Xe lamp	0.5	3.8	[23]
**HCCN-2-Pt**	1.22 wt.% Pt	TEOA	300 W Xe lamp	8.67	20.1	[24]

Supplementary References

[1] T. D. Kuhne, M. Iannuzzi, M. Del Ben, V. V. Rybkin, P. Seewald, F. Stein, T. Laino, R. Z. Khaliullin, O. Schutt, F. Schiffmann, D. Golze, J. Wilhelm, S. Chulkov, M. H. Bani-Hashemian, V. Weber, U. Borstnik, M. Taillefumier, A. S. Jakobovits, A. Lazzaro, H. Pabst, T. Muller, R. Schade, M. Guidon, S. Andermatt, N. Holmberg, G. K. Schenter, A. Hehn, A. Bussy, F. Belleflamme, G. Tabacchi, A. Gloss, M. Lass, I. Bethune, C. J. Mundy, C. Plessl, M. Watkins, J. VandeVondele, M. Krack, J. Hutter, *J. Chem. Phys*. **2020**, 152, 194103.

[2] S. Goedecker, M. Teter, J. Hutter, *Phys. Rev. B Condens. Matter* **1996**, 54, 1703.

[3] C. Hartwigsen, S. Goedecker, J. Hutter, *Phys. Rev. B* **1998**, 58, 3641.

[4] M. Krack, *Theor. Chem. Acc.* **2005**, 114, 145.

[5] J. VandeVondele, J. Hutter, *J. Chem. Phys.* **2007**, 127, 114105.

[6] S. Grimme, J. Antony, S. Ehrlich, H. Krieg, *J. Chem. Phys.* **2010**, 132, 154104.

[7] S. Grimme, S. Ehrlich, L. Goerigk, *J. Comput. Chem.* **2011**, 32, 1456.

[8] J. Moellmann, S. Grimme, *J. Phys. Chem. C* **2014**, 118, 7615.

[9] M. K. Horton, P. Huck, R. X. Yang, J. M. Munro, S. Dwaraknath, A. M. Ganose, R. S. Kingsbury, M. Wen, J. X. Shen, T. S. Mathis, A. D. Kaplan, K. Berket, J. Riebesell, J. George, A. S. Rosen, E. W. C. Spotte-Smith, M. J. McDermott, O. A. Cohen, A. Dunn, M. C. Kuner, G. M. Rignanese, G. Petretto, D. Waroquiers, S. M. Griffin, J. B. Neaton, D. C. Chrzan, M. Asta, G. Hautier, S. Cholia, G. Ceder, S. P. Ong, A. Jain, K. A. Persson, *Nat. Mater.* **2025**, 24, 1522.

[10] Q. Han, B. Wang, J. Gao, Z. Cheng, Y. Zhao, Z. Zhang, L. Qu, *ACS Nano* **2016**, 10, 2745.

[11] J. Ran, W. Guo, H. Wang, B. Zhu, J. Yu, S. Z. Qiao, *Adv. Mater.* **2018**, 30, e1800128.

[12] Q. Zhu, B. Qiu, M. Du, J. Ji, M. Nasir, M. Xing, J. Zhang, *ACS Sustain. Chem. Eng.* **2020**, 8, 7497.

[13] Q. Han, Z. Cheng, B. Wang, H. Zhang, L. Qu, *ACS Nano* **2018**, 12, 5221.

[14] B. Yan, C. Du, G. Yang, *Small* **2020**, 16, e1905700.

[15] Y. Chen, X. Liu, L. Hou, X. Guo, R. Fu, J. Sun, *Chem. Eng. J.* **2020**, 383, 123132.

[16] N. Meng, J. Ren, Y. Liu, Y. Huang, T. Petit, B. Zhang, *Energy Environ Sci.* **2018**, 11, 566.

[17] Y. Yu, W. Yan, X. Wang, P. Li, W. Gao, H. Zou, S. Wu, K. Ding, *Adv. Mater.* **2018**, 30, 1705060.

[18] H. Schlomberg, J. Kroger, G. Savasci, M. W. Terban, S. Bette, I. Moudrakovski, V. Duppel, F. Podjaski, R. Siegel, J. Senker, R. E. Dinnebier, C. Ochsenfeld, B. V. Lotsch, *Chem. Mater.* **2019**, 31, 7478.

[19] F. Gao, H. Xiao, J. Yang, X. Luan, D. Fang, L. Yang, J. Zi, Z. Lian, *Appl. Catal. B-Environ.* **2024**, 341, 123334.

[20] Q. Deng, G. Ba, T. Huo, H. Li, W. Hou, *Appl. Catal. A-Gen.* **2020**, 606, 117833.

[21] J. Kröger, A. Jiménez-Solano, G. Savasci, P. Rovó, I. Moudrakovski, K. Küster, H. Schlomberg, H. A. Vignolo-González, V. Duppel, L. Grunenberg, C. B. Dayan, M. Sitti, F. Podjaski, C. Ochsenfeld, B. V. Lotsch, *Adv. Energy Mater.* **2020**, 11, 2003016.

[22] C. Lv, W. Li, Q. Lin, H. Yang, Y. Jiang, Y. Yu, Y. Huang, *J. Mater. Chem. A* **2023**, 11, 20701.

[23] D. Feng, Y. Cheng, J. He, L. Zheng, D. Shao, W. Wang, W. Wang, F. Lu, H. Dong, H. Liu, R. Zheng, H. Liu, *Carbon* **2017**, 125, 454.

[24] Y. Pang, L. Li, Q. Bu, R. Zhang, Y. Lin, T. Xie, *Adv. Funct. Mater.* **2025**, 35, 2501108.
